# sigFeature: Novel Significant Feature Selection Method for Classification of Gene Expression Data Using Support Vector Machine and *t* Statistic

**DOI:** 10.3389/fgene.2020.00247

**Published:** 2020-04-03

**Authors:** Pijush Das, Anirban Roychowdhury, Subhadeep Das, Susanta Roychoudhury, Sucheta Tripathy

**Affiliations:** ^1^Computational Genomics lab, Structural Biology and Bioinformatics Division, CSIR- Indian Institute of Chemical Biology, Kolkata, India; ^2^Department of Oncogene Regulation, Chittaranjan National Cancer Institute, Kolkata, India; ^3^Saroj Gupta Cancer Centre and Research Institute, Kolkata, India; ^4^Academy of Scientific and Innovative Research, New Delhi, India

**Keywords:** feature selection, machine learning, support vector machine, microaaray, cancer, RNA-Seq, bootstrap, GSEA

## Abstract

Biological data are accumulating at a faster rate, but interpreting them still remains a problem. Classifying biological data into distinct groups is the first step in understanding them. Data classification in response to a certain treatment is an extremely important aspect for differentially expressed genes in making present/absent calls. Many feature selection algorithms have been developed including the support vector machine recursive feature elimination procedure (SVM-RFE) and its variants. Support vector machine RFEs are greedy methods that attempt to find superlative possible combinations leading to binary classification, which may not be biologically significant. To overcome this limitation of SVM-RFE, we propose a novel feature selection algorithm, termed as “sigFeature” (https://bioconductor.org/packages/sigFeature/), based on SVM and *t* statistic to discover the differentially significant features along with good performance in classification. The “sigFeature” R package is centered around a function called “sigFeature,” which provides automatic selection of features for the binary classification. Using six publicly available microarray data sets (downloaded from Gene Expression Omnibus) with different biological attributes, we further compared the performance of “sigFeature” to three other feature selection algorithms. A small number of selected features (by “sigFeature”) also show higher classification accuracy. For further downstream evaluation of its biological signature, we conducted gene set enrichment analysis with the selected features (genes) from “sigFeature” and compared it with the outputs of other algorithms. We observed that “sigFeature” is able to predict the signature of four out of six microarray data sets accurately, whereas the other algorithms predict less data set signatures. Thus, “sigFeature” is considerably better than related algorithms in discovering differentially significant features from microarray data sets.

## Introduction

Feature selection is described as a process in which a subset of relevant features is selected from a larger data set. These features are used for model construction. On the basis of the model created, one can separate or classify the classes present in a data set (James et al., 2013). Based on the combination of selection algorithms and model building, feature selection methods are normally classified into three classes such as Filter methods, Wrapper methods, and Embedded methods. All the feature selection algorithms used in this article belong to Wrapper methods. A subset of features is used in Wrapper methods, and a model is trained with those features. The user then decided to add or remove features from the subset based on the inferences from the previous model. Generally, microarray data sets are high-dimensional in nature. It is obvious that not all the features contribute to the prediction variable. Removing low-importance features will not only increase the accuracy but also reduce the complexity of the model and its overfitting. Therefore, the training time for the very large data sets can also be reduced. Usually, the feature selection algorithms (Allison et al., 2006) are used for different tasks such as classification, clustering, and regression analysis (Jović et al., 2015). Feature selection algorithm is widely used in analyzing different types of biological data set, for example, whole-genome expression data set (Ramaswamy et al., 2001; Frank et al., 2006; Zheng et al., 2006), protein mass spectra data set (Hilario et al., 2006), whole-genome sequencing data set (Das et al., 2006), and so on.

Differential expression analysis technologies on different data sets such as RNAseq and microarrays provide a great opportunity for the researchers to quantify the expression levels of thousands of cellular genes concurrently. This advantage led to its rampant use in clinical settings. A general approach is to employ *t* statistic (along with multiple hypothesis tests) on microarray data sets to obtain the statistically significant differentially expressed genes (DEGs). However, the DEGs produced by most of the methods often contain biologically irrelevant data lacking discriminatory power to classify the groups present in the data set. This is also the same with the clinical data. It is therefore essential to develop an appropriate algorithm to identify biologically significant DEGs together with excellent classification precision in a tumor versus normal tissue contrast (Galland et al., 2010) or in distinct tumor subtypes (Bonome et al., 2008).

Over the past few years, many feature selection algorithms have been developed for microarray data analysis. However, most of the binary (two-class) feature selection algorithms focus mostly on classification accuracy of the selected features, which often fails to be biologically relevant leading to inaccurate downstream data analysis (Golub et al., 1999; Guyon et al., 2002; Lee et al., 2003; Zhang et al., 2006a,b; Zhou and Mao, 2005; Li et al., 2012; Mishra and Mishra, 2015). So, a better classifier that has the ability to select features with greater discriminatory power and more biological insight is required for clinical practice (Roepman et al., 2005, 2006). We have used expression values of different cancer types [e.g., breast cancer (GSE3744), oral cancer (GSE25099), ovarian cancer (GSE26712), squamous cell carcinoma (GSE2280), lung cancer (GSE7670), and brain cancer (GSE4290)] to test and compare “sigFeature” algorithm with other algorithms.

In this study, we have created a novel feature selection algorithm “sigFeature” that is based on support vector machine (SVM) and *t* statistic. Our algorithm not only selects features with higher classification accuracy but also determines the differentially expressed features (genes) seamlessly. After comparing “sigFeature” with three selection algorithms such as “SVM-RFE,” “SVM-T-RFE,” and “SVM-BT-RFE” (Supplementary Data Sheet 1) using the aforementioned data sets, “sigFeature” stands apart in terms of feature classification, as well as differentially expressed features in almost all the classes studied. We further tested “sigFeature” for determining its ability to predict the biological signatures of the example data sets. It was found that when gene set enrichment analysis (GSEA) analysis (Subramanian et al., 2005) was done on the output of “sigFeature,” the biological features of the samples were predicted accurately. “sigFeature” is proven to be better than other algorithms when tested with GSEA with the selected features.

## Materials and Methods

We have chosen six publicly available microarray data sets randomly for testing and comparison with our tool. All the feature selection algorithms are applied precisely for cancer classification. In addition to most feature selection methods embedded with an estimation of the classifier, “sigFeature” is inherently a multivariate method. “sigFeature” evaluates the relevance of several features considered together. A univariate method, on the other hand, assesses the significance of each feature individually. The latter is often computationally easier, but the former is more sophisticated from the data analysis point of view, as genes are known to communicate in many respects and are often coregulated. We also used the selection approach of the ensemble feature that relies on various subsamples of the original data to create different signatures. Using GSEA analysis, the selected robust signatures are finally analyzed to determine its biological signature.

### Microarray Data Sets

Table 1 summarizes the main characteristics of the microarray data sets downloaded from GEO (Gene Expression Omnibus). The data sets share common characteristics such as very low samples/dimensions (or features) ratio.

**Table 1:** Description of the data sets.

**Data set**	**Samples**	**Probes**	**Classes**	**SDR**	**Types**	**Reference**
GSE3744	47	54675	2	0.00086	Breast cancer	Richardson et al., 2006
GSE25099	79	17881	2	0.00441	Oral cancer	Peng et al., 2011
GSE26712	195	22283	2	0.00875	Ovarian cancer	Bonome et al., 2008
GSE2280	27	22283	2	0.00121	Squamous cell carcinoma	O'Donnell et al., 2005
GSE7670	66 (54^*^)	22283	2	0.00296	Lung cancer	Su et al., 2007
GSE4290	180	54613	2	0.00329	Brain cancer	Sun et al., 2006

* Total 27 paired samples are used in this example.

*Samples/dimensions ratio (SDR) refers to the ratio between the number of samples and the number of dimensions (or features)*.

All these data sets are preprocessed using the following procedures.

### Data Normalization

The purpose of data normalization is to minimize the variation in data due to various non-biological factors and make them comparable in one scale. These data sets are normalized by “quantile” normalization method using the “Bioconductor” package “Limma” (Ritchie et al., 2015). The goal of the “quantile” normalization method (Bolstad et al., 2003) is to make the distribution of sample intensities equal for each array in a set of arrays.

### Support Vector Machines

Generally, SVMs are used for classification and regression analysis as supervised learning methods. Support vector machines are also used for selecting features from a data set [e.g., SVM recursive feature elimination (SVM-RFE)]. For feature selection, we have used SVM in “sigFeature,” with some modification to obtain the desired weight values for each feature. In this section, we have explained how SVM effectively operates, because our algorithm is built upon the basic principle of SVM.

Support vector machine uses kernel functions to perform classification efficiently on non-linear data. It implements implicit mapping in the high-dimensional input feature vectors into high-dimensional spaces. This produces a linear hyperplane, which can separate two groups of data meaningfully (Butte, 2002). The hyperplane in higher dimensional spaces is chosen to be maximally distant from both groups so that examples from separate classes are separated as much as possible. New examples are then classified based on their orientation to the hyperplane. The hyperplane can be expressed as a vector (Equation 1, linear SVM) that acts as a reference frame to map the position of each sample in higher dimensional spaces and is summed to yield a discriminate score, which is used to classify the sample into one of the two groups.

(1) $$\begin{array}{r} f\left(x\right)=\overset{d}{\underset{j=1}{\sum }}w_{j}x_{j}+b \end{array}$$

Assuming a training data set {(*x*1, *y*1), …, (*xn, yn*), $x_{i}\in {ℜ}^{d}$, *y*_*i*_ ∈ (−1, 1)}, where *x* = (*x*_1_, …, *x*_*d*_) is a *d-*dimensional input vector, and *y* is the class label, and *w* = (*w*_1_, …, *w*_*d*_) are coefficients of the hyperplane, and *b* represents the intercept of the hyperplane. Support vector machines are used in different feature selection algorithms, for example, “SVM-RFE,” “SVM-T-RFE,” and “SVM-BT-RFE.” A detailed description of those algorithms is given in the Supplementary Data Sheet 1.

### Support Vector Machine–Recursive Feature Elimination

In 2002, Guyon et al. (2002) introduced a feature selection method known as SVM-RFE for classification of cancer. The “SVM-RFE” is a weight-based method. The weight vector coefficients of a linear SVM are applied at each stage as a feature ranking criterion. The most informative features are those that correspond to the largest weight. Thus, a sequential backward feature elimination procedure is used by “SVM-RFE” for selecting the feature with the smallest weight that is subsequently stored into a stack. This iteration process is continued until the last feature variable remains. The “SVM-RFE” algorithm and our newly developed feature selection algorithm both belong to the Wrapper method, and both algorithms select the feature by eliminating feature recursively. The main difference is there in calculating the ranking score for the *i*th feature.

### Significant Feature Selection (sigFeature)

“SVM-RFE” (Guyon et al., 2002) algorithm was originally written for selecting features in binary classification problem. Some researchers (Li et al., 2012; Mishra and Mishra, 2015) enhanced “SVM-RFE” for further betterment of the feature selection process. Li et al. (2012) and Mishra and Mishra (2015) used different mathematical equitation in their algorithms to calculate the ranking score for the *i*th feature. For more information, see Supplementary Data Sheet 1.

Generally, the expression data set contains a large number of probes sets and a small number of sample sizes. It is observed that the number of samples in each class is unequal in the data set. In our approach, the features to be selected for classification need to contain maximum discriminatory power between the classes. Considering the imbalance in sample size (no. of samples class 1 ≠ class 2) into consideration, we characterize the accompanying measure as below (Equation 2):

(2) $$\begin{array}{r} \Delta \delta =\frac{1}{n^{+}}\underset{x^{+}\in class1}{\sum }f\left(x^{+}\right)-\frac{1}{n^{-}}\underset{x^{-}\in class2}{\sum }f\left(x^{-}\right) \end{array}$$

In class +1 and −1, the total number of samples is considered as *n*^+^ and *n*^−^, respectively. The value Δδ denotes the separation between the two classes. The larger the value of Δδ, the better is the separation between the two classes. Considering equation (2) and denoting the difference of the *j*th feature of the two classes such as $\left|t_{j}^{+}\right|$ and $\left|t_{j}^{-}\right|$, we get Equation 3:

(3) $$\begin{array}{r} \Delta \delta =\overset{d}{\underset{j=1}{\sum }}w_{j}|t_{j}^{+}|-\overset{d}{\underset{j=1}{\sum }}w_{j}|t_{j}^{-}|=\overset{d}{\underset{j=1}{\sum }}w_{j}\left(\left|t_{j}^{+}|-|t_{j}^{-}\right|\right) \end{array}$$

The *j*th element of *w* (weight vector) is considered, as *w*_*j*_ and *d* is the total number of features. In special cases, where one class has only one sample, and the other class has more than one sample, ***t***, the significant differences between the two classes can be calculated by using standard single sample *t* statistic where $\left|t_{j}^{+}\right|$ stands for class +1 and $\left|t_{j}^{-}\right|$ stands for class −1.

(4) $$\begin{array}{r} \text{where}|t_{j}^{+}|=|\frac{\left({{\bar{x}}_{j}}^{+}-\mu_{j}^{+}\right)}{\sqrt{{\left(s_{j}^{+}\right)}^{2}/n^{+}}}|and|t_{j}^{-}|=|\frac{\left({\bar{x}}_{j}^{-}-\mu_{j}^{-}\right)}{\sqrt{{\left(s_{j}^{-}\right)}^{2}/n^{-}}}| \end{array}$$

where ${\bar{x}}_{j}^{+}$ and ${\bar{x}}_{j}^{-}$ stands for mean, $s_{j}^{+}$ and $s_{j}^{-}$ are standard deviation, $\mu_{j}^{+}$ and$\mu_{j}^{-}$ stand for the specified population mean of the *j*th feature, and *n*^+^ and *n*^−^ are the number of samples present in each class (+1 class and −1 class), respectively.

(5) $$\begin{array}{r} \Delta \delta =w_{j}*\left(\left|t_{j}^{+}|-|t_{j}^{-}\right|\right) \end{array}$$

$X_{0}={\left[x_{1},x_{2},\ldots ..x_{k},\ldots x_{l}\right]}^{T}$ If both classes have more than one sample, then the separation value Δδ can be calculated by the equation as below:

(6) $$\begin{array}{r} \Delta \delta =w_{j}*\left|\frac{\left({\bar{x}}_{j}^{+}-{\bar{x}}_{j}^{-}\right)}{\sqrt{\left({\left(s_{j}^{+}\right)}^{2}/n^{+}\right)+\left({\left(s_{j}^{-}\right)}^{2}/n^{-}\right)}}\right| \end{array}$$

From the above equation, the maximum separation would be affected when the expression values of an individual feature in both classes reject the null hypothesis **Ho** and move on in the direction of a specified alternative hypothesis **Ha**.

(7) $$\begin{array}{r} \Delta \delta =w_{j}*P\left[t_{j}\left(v\right)<-|u|ort_{j}\left(v\right)>|u|\right] \end{array}$$

where ***v*** is the level of flexibility parameter for the corresponding reference distribution, and ***u*** is the observed (positive) estimation of the test statistic on the basis of the level of flexibility ***v***. Unlike “SVM-RFE,” in “sigFeature” we select important features by incorporating the product of weights and the corresponding differences between the classes. The pseudo code of the algorithm is given as below.

**Algorithm 1:** sigFeature

Inputs: Training examples $X_{0}={\left[x_{1},x_{2},\ldots ..x_{k},\ldots x_{l}\right]}^{T}$ Class labels $y={\left[y_{1},y_{2},\ldots ..y_{k},\ldots y_{l}\right]}^{T}$ Initialize: Subset of existing features *s* = [1, 2, …*n*] Feature ranked list *r* = [] Repeat until *s* = [] Confine training examples to good feature inventories *X* = *X*_0_(:, *s*) Train the classifier (training data set) α = *SVM* − *train* (*X, y*) Enumerate the weight vector of dimension length(s) $W=\underset{k}{\sum }\alpha_{k}y_{k}x_{k}$ Enumerate the ranking criteria (for all *i*) *c*_*i*_ = *w*_i_*P[*t*_*i*_(*v*) < −|*u*|*ort*_*i*_(*v*) > |*u*|] Find the feature with the negligible ranking criterion *f* = *sort*(*c*) Reform feature ranked list: *r* = [*s*(*f*), *r*] Remove the feature with the smallest ranking criterion *s* = *s*(1:*f* − 1, *f* + 1:*length*(*s*)) Output: Feature ranked list *r*.

### Robust Selection of Features Using the Ensemble Selection Procedure

We have further embedded the recent concept of ensemble feature selection techniques to improve the stability of feature selection algorithms (Abeel et al., 2010). Like ensemble learning for classification, the technique of selection of features uses the same idea. A number of different selectors of features are used in this method (ensemble), and finally, the output of these separate selectors is aggregated and returned as the final result. Using our newly developed algorithm “sigFeature,” we focus on the analysis of ensemble feature selection technique to select robust features. The other feature selection algorithms (“SVM-RFE,” “SVM-T-RFE,” and “SVM-BT-RFE”) included in this work for performance comparison with “sigFeature” select robust features in a similar way.

We chose our training set with 40 subsampling sets consisting of 90% of the original data set. The remaining 10% of the data can be used to evaluate classification performance as an independent validation set. The main motto behind this is to generate a variety of feature selection protocols using “sigFeature” algorithm. Because the “sigFeature” algorithm is deterministic, performing it on different training samples is the only way to generate diversity in selection. To this end, we use the bootstrapping method, a well-established statistical technique for reducing variance (Efron, 1992). In statistics, bootstrap is a method that relies on random sampling with replacement. Within each layer, a simple random sample is selected from the n−1 clusters within the n clusters of the layer. The process can be repeated “N” times by producing N new samples. Bootstrap weights are generated in each layer for infinite populations (“with replacement” sampling) by sampling with replacement from the primary sampling unites. The weights of the bootstrap are used to measure values of “N” that are used to determine the variance. By drawing different bootstrap samples of the training data (with replacement), we can apply “sigFeature” algorithm to each of these bootstrap samples and thus obtain a variety of feature rankings. Then, we have an ensemble (Ensemble feature selection) composed of *t* feature selectors, EFS = *{F1,F2,…Ft}*, so we assume that each *Fi* gives the ranking feature, $f_{i}=\left(f_{i}^{1},\ldots ,f_{i}^{N}\right)$, where $f_{i}^{j}$ indicates the rank of feature *j* in bootstrap *i*. Rank 1 is assigned for the best feature, and the worst is ranked N. In order to aggregate the various rankings obtained by bootstrapping the training data, we have chosen a complete linear aggregation (CLA) in a final signature. A general formula obtained by summing the ranks over all bootstrap samples for the ensemble ranking *f* is as follows:

(8) $$\begin{array}{r} f=\left(\overset{t}{\underset{i=1}{\sum }}w_{i}f_{1}^{1},\ldots ..,\overset{t}{\underset{i=1}{\sum }}w_{i}f_{1}^{N}\right) \end{array}$$

where *w*_*i*_ denotes a bootstrap-dependent weight.

Then, the ensemble ranking *f* is obtained by simply summing the ranks above all samples of the bootstrap.

The *s* features with the highest summed rank are selected from *f* to select the final set of features for a size *s* signature.

### Stability Measurement of the Selected Features

Stability measurement is an essential part of the classification performance. It is therefore necessary to incorporate stability, else any feature selection algorithm may always return the same features regardless of any input sample combinations. We used Kuncheva index (KI) statistical analysis described below to measure stability of the selected features.

Let us consider a data set *X* = {*x*_1_, …., *x*_*M*_} with M samples and N features. After that, the sample set is subsampled randomly with *k* times of size [*xM*](0 < *x* < 1). We randomly selected *k* = 40 in our data analysis and *x* = 0.9. Consequently, on each of the k subsamplings, feature selection is performed, and a marker set of a given size is selected, further referred to as a signature. The relative stability *S*_*tot*_ can then be defined as the average of all pair-like comparisons between all the signatures on the *k* subsamplings.

(9) $$\begin{array}{r} S_{tot}=\frac{2\sum_{j=1}^{k}\sum_{j=i+1}^{k}KI\left(f_{i},f_{j}\right)}{k\left(k-1\right)} \end{array}$$

where *f*_*i*_ is the signature obtained by the subsampling method *i*(1 ≤ *i* ≤ *k*). *KI*(*f*_*i*_, *f*_*j*_) denotes the KI. A stability index defined as follows between *f*_*i*_ and *f*_*j*_ (Kuncheva, 2007).

(10) $$\begin{array}{r} KI\left(f_{i},f_{j}\right)=\frac{rN-s^{2}}{s.\left(N-s\right)}=\frac{r-\left(s^{2}/N\right)}{s-\left(s^{2}/N\right)} \end{array}$$

where *s* = |*f*_*i*_| = |*f*_*j*_| refers to the signature size, and *r* = |*f*_*i*_⋂*f*_*j*_| refers to the number of common elements in both signatures. The KI meets −1 < *KI*(*f*_*i*_, *f*_*j*_) ≤ 1, and the higher its value, the greater the number of features commonly selected in both signatures. In this index, the *s*^2^/*N* term corrects a bias due to the chance to select common features between two randomly selected signatures.

### Classification Performance (Cross-Validation)

In 2002, Ambroise and McLachlan (2002) introduced a new cross-validation technique for evaluation of feature (gene) selection algorithms. In this technique, the feature selection is performed on a subset of samples, which are obtained from the total sample sets, and the validation is performed on the other subsample set to acquire an impartial execution test. In this research work, we recruited external 10-fold cross-validation data set to assess the performance of the selected features.

### GSEA for Prediction of Biological Signature in the Test Data Sets

An important attribute of a selected feature is its potential to predict the biological signature of any data set. In GSEA, we used the Molecular Signatures Database (MSigDB V 7.0) to compare selected features using the aforementioned algorithms for further downstream evaluation. MSigDB V 7.0 is a compilation of annotated gene sets. For this analysis, Cancer Gene Neighborhoods (CGN), Cancer modules (CM), and Oncogenic signatures (C6) gene sets were used during GSEA (Subramanian et al., 2005), with the first 500 selected features generated by each of the algorithms including “sigFeature.” Briefly, CGN is a collection of gene sets defined by expression neighborhoods centered on 380 cancer associated genes, whereas CM is an assemblage of 456 gene set “modules” observed to significantly change during various oncogenic conditions (Segal et al., 2004), and finally, C6 is a collection of gene sets (oncogenic signatures, 189 gene sets) that represent signatures of biological pathways that are generally deregulated in carcinogenesis.

### Implementation and Availability

All the algorithms (“sigFeature,” “SVM-RFE,” “SVM-T-RFE,” and “SVM-BT-RFE”) are implemented in R script. The “sigFeature” package is available in Bioconductor and GitHub repository (https://bioconductor.org/packages/sigFeature). We use SVM by using a CRAN package named “e1071” (Meyer et al., 2018). The R script for the algorithm “SVM-RFE” (Guyon et al., 2002) is publicly available. Because there are no fast implementations of “SVM-T-RFE” and “SVM-BT-RFE” accessible so far, we used the “e1071” package to implement these two algorithms in R.

## Results

**Figure 1:**
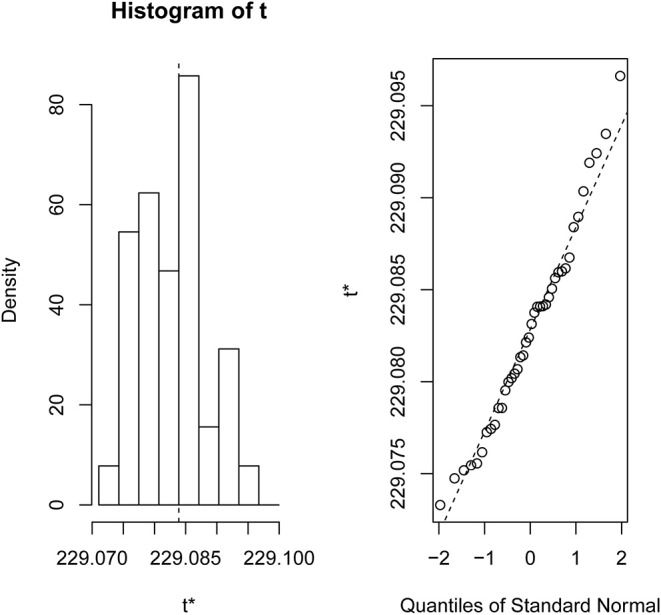
Bootstrap distribution plot and normal Q-Q plot for the data set GSE2280. We used 40 bootstraps (with replacement) with 90% of the total samples (GSE2280) to randomize the array of subsamples.

The experimental evaluations on the six different types of cancer microarray data sets have been reported here. We also analyzed the RNA-Seq data set (Canine mammary gland tumors, GSE119810) for reestablishing the robustness of the newly developed algorithm (detailed results are available in Supplementary Data Sheet 3). Finding a robust feature list (Roepman et al., 2005) from a large sample pool without any sample biasness is a very tedious work. In order to compare the list of features produced by “sigFeature” algorithm with the features obtained from other algorithms discussed (e.g., “SVM-RFE,” “SVM-T-RFE,” “SVM-BT-RFE”), a comparable model for the selection method needs to be followed. We resampled 90% of the total data using bootstrap method (with replacement), and the resampling was done 40 times (bootstrapping graphics is shown in Figure 1). The remaining 10% samples are used to evaluate the features selected. For the assessment of the combined significance of marker sets, the latest classification performance has already been provided. The results show that both stability and accuracy of the classification are quite significant on the data set compared to stability measurement and classification of the selected features of other algorithms such as “SVM-RFE,” “SVM-T-RFE,” and “SVM-BT-RFE.” The results of the variance in pairwise stability between two signatures are also discussed. The complementary performance of the various feature selection algorithms of consensus building from different individual signatures is carefully evaluated. We performed the *t*-test of the selected features individually between the classes in the data set to see the significant difference of the groups. The features that are more distinctly different will assist further downstream assessment. Thus, the GSEA is used to observe the contribution by using these robust features in discovering the cancer signature.

### Stability of the Selected Features

**Figure 2:**
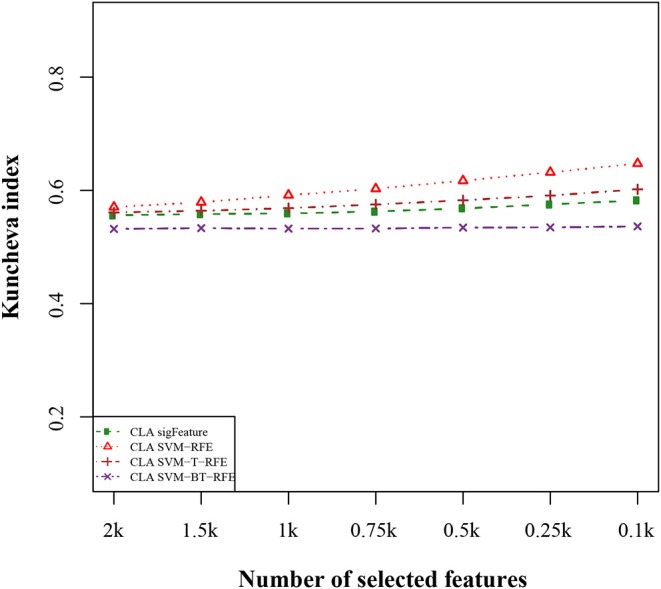
Kuncheva index plot for the data set GSE2280. The stability of the features is measured based on CLA methods for different feature selection algorithms. We used 40 bootstraps (with replacement) and eliminated *E* = 1% features.

**Figure 3:**
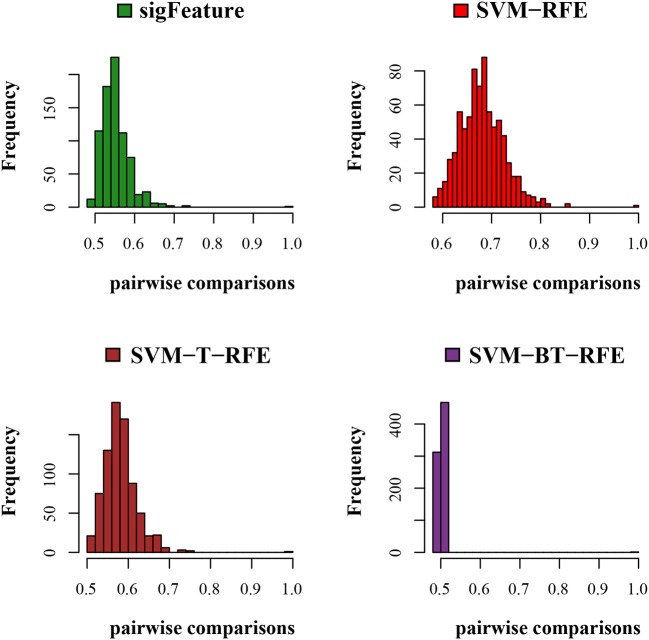
Histogram plots for pairwise stability comparison of the features. Distribution plots of the pairwise stabilities for the data set GSE2280 where different algorithms produce the feature lists. In each iteration of the algorithms, we used 40 bootstraps, eliminated *E* = 1% features, used a signature size of 10%, and selected the CLA aggregation model.

We compared the feature of each of the algorithms and analyzed the stability of each of the six cancer data sets used in this study. Figure 1 shows the results of the data set GSE2280 using a default configuration with the bootstrap number of 40, and all feature selection algorithms have been applied, with *E* = 1% at each iteration. Results of the other data sets are represented in Supplementary Figures (Figure 2.1 to Figure 6.4.2 in Supplementary Data Sheet 2). To minimize the execution time taken by each algorithm, the default configuration is used. The KI measures the robustness of the selected signatures (marker sets), and the external cross-validation error is used to measure the classification performance (Figure 2). Kuncheva index is generally calculated between two feature vectors. The more the KI is closer to 1, the more the vectors are similar. It can be observed in the literature (Kuncheva, 2007) that the CLA methods significantly improve the baseline (only SVM-RFE) in terms of both stability and classification performance. Methods of ensemble are better able to eliminate noisy and irrelevant dimensions. So we chose to produce the robust feature using the CLA method in our present study. One important issue in stable feature selection is how to measure the “stability” of feature selection algorithms, that is, how to qualify selection sensitivity for training set variations. The measure of stability can be used in various contexts. On the one hand, evaluation of various algorithms in performance comparison is indispensable. On the other hand, in feature selection algorithms, which take stability into account, it can be used for internal validation. We have chosen a feature list as a reference list in our steady measurement experiment, and the remaining feature lists are measured based on the reference feature list. Finally, the stability value of pairwise experiment is represented as a plot of histogram (Figure 3).

### Classification Performance Results

**Figure 4:**
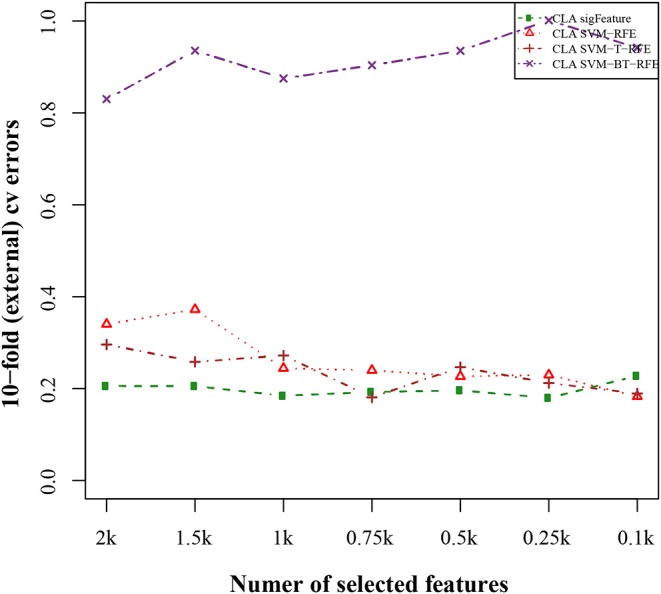
Tenfold external cross-validation error plot. The classification performances of the top features are shown here, which are selected by different feature selection algorithms. We used 40 bootstraps and eliminated *E* = 1% features.

**Figure 5:**
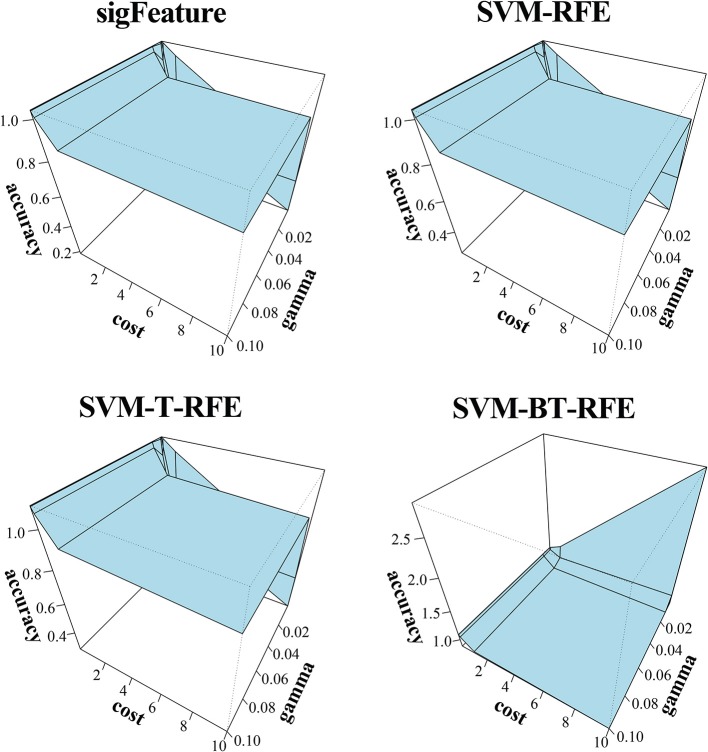
Three-dimensional representation of cost, gamma, and classification accuracy. The Cost and gamma values are selected to determine the best performance in the classification of features selected (top 1,000 features by CLA) by different selection algorithms.

The results of the classification of the GSE2280 microarray data set are illustrated here, and outputs for other data sets are included in Supplementary Data Sheet 2. We used different publicly available algorithms (“SVM-RFE,” “SVM-T-RFE,” and “SVM-BT-RFE”) to compare the performance of the selected feature with “sigFeature” for this data set. From the 90% of the total data set, the feature lists are selected, and the 10% of samples are used for testing the selected feature. We also subsampled by iterating 40 times the 90% of the total samples using the bootstrapping (with replacement) method to remove the sample biasness. After that, we use the improved ensemble selection method (“CLA”) to find out the robust feature list. Using the feature list, the classification performance is tested on the test sample set. The graphical representation of the result is shown in Figure 4. In order to find the best classification accuracy, we have adjusted the parameters cost and gamma (Figure 5). The gamma parameter intuitively defines the extent to which the influence of a single example of training reaches, with low values meaning “far” and high values meaning “close.” The cost parameter offers proper classification of training examples to maximize the decision function margin. The “SVM-BT-RFE,” an individual ranking method, performs more badly than other algorithms evaluated in this study because this algorithm selects many redundant features, which give minimal preferential power to the classification issue. The classification performance here depends on many conditions. The major factors are the number of samples in a data set and the numeric values corresponding to the expression of each gene in that data set.

### Differentially Significant Features

**Figure 6:**
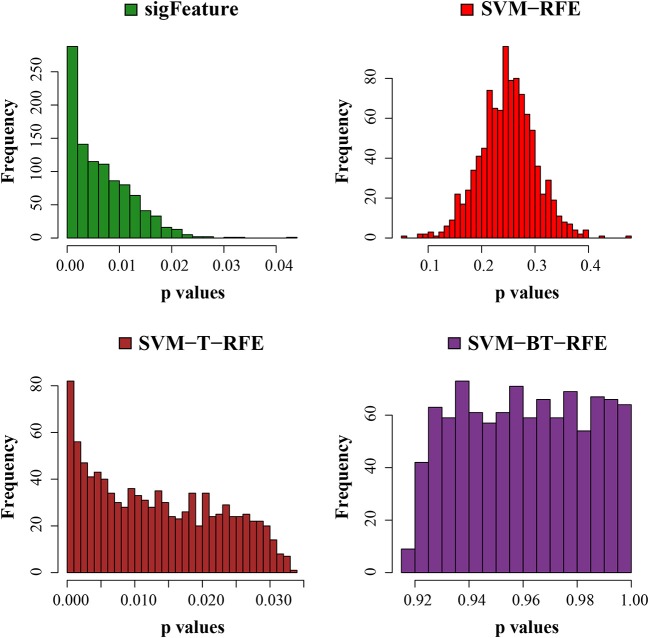
Histogram plots of unadjusted *p*-values. The comparison of the average unadjusted *p*-values is shown, which are calculated individually using the top 1,000 features between classes. The list of features is made using 40 bootstrap subsets where the feature selection algorithms remove *E* = 1% features at each iteration.

In order to obtain a meaningful biological insight, the selected features must have a significant difference between the sample groups present in the data sets. In this experiment, we compare the characteristics (significant difference between the groups) of the features produced by “sigFeature” with other algorithms. Thus, we compare the *p*-values of each feature (top *n* = 1,000), which are produced by the different algorithms. Although individual feature lists are produced by each subset (40 bootstraps), we average the *p*-value by its position in the feature list. In each data set, it may not create an equal number of sample size in both classes. We use default *t*-test (two-sided) using an R function named *t*-test()and computed the unadjusted *p*-values. Then the unadjusted *p*-values produced are used to plot a histogram, depicted in Figure 6 to observe the frequency of the unadjusted *p*-values associated with the rank feature. The histogram plot shows the area proportional to the unadjusted *p*-value frequency.

### GSEA Results

The features identified by newly developed “sigFeature” (top five hundred features by CLA) and other three algorithms SVM-RFE, SVM-T-RFE, and SVM-BT-RFE were analyzed by the aforementioned workflow to check whether they are able to predict the biological attribute of the six microarray data sets from which they were derived.

The first data set GSE2280 is a critical data set as it originates from oral cancer patients with lymphatic metastasis. Interestingly, “sigFeature” was able to significantly (*p* ≤ 0.05) predict two gene sets “Neighborhood of RAP1A” and “Neighborhood of UBE2N.” Both genes were strongly involved in regulation of tumor metastasis. The monomeric G protein, RAP1A, acts as a switch during transduction of cellular signaling and generally regulated by its binding to either guanosine triphosphate (GTP) or guanosine diphosphate. It functions to regulate the function of cell adhesions and junction proteins and mediate cellular migration and polarization to promote metastasis in prostrate tumors, ovarian tumors, melanoma, lung cancer, glioma, bladder cancer, leukemia, and also oral cavity (Bailey et al., 2009; Chen et al., 2013; Lu et al., 2016; Yi-Lei et al., 2017). Moreover, UBE2N encodes a protein that is a member of the E2 ubiquitin–conjugating enzyme family and helps to catalyze the synthesis of non-canonical “Lys-63”-linked polyubiquitin chains leading to transcriptional activation of genes involved in tumor proliferation and metastasis (Gallo et al., 2017; Vallabhaneni et al., 2017). Among the other algorithms, SVM-RFE only enriched the term “Neighborhood of RAN” that is related to signature of cancer metastasis. RAN is also a GTPase, which regulates the nucleocytoplasmic import and export of proteins and RNAs, and reported to be involved in the metastasis of renal cell carcinoma and pancreatic cancer (Abe et al., 2008; Deng et al., 2014). No other algorithms were able to identify such intricate aspect in the given data set.

Data set GSE4290 is derived from glioma patients. The feature identified by “sigFeature” was able to enrich computational gene sets “Genes in the cancer module 83” and “Genes in the cancer module 151.” Interestingly, these modules were composed mostly of data sets derived from primary neuro tumors and related cell lines. Hence, it appears that “sigFeature” is able to partially identify its biological attributes correctly compared to other algorithms that failed to do so.

In case of data set GSE7670, expression of genes was obtained from lung cancer samples and cell lines. Feature selected by only SVM-T-RFE was not able to predict its biological nature. Other algorithms along with “sigFeature” are able to predict its true biological origin by enrichment of oncogenic signature (C6) “Module_5 Lung genes.”

Data set GSE26712 is based on ovarian cancer. Feature selected by “sigFeature” also predicted its biological origin with the enrichment of oncogenic signature (C6) “Module_1 Ovary genes.” Among the other algorithms, only SVM-RFE–derived feature was able to predict that the data set is of ovarian origin by enrichment of the same module.

Data set GSE3744 is composed of breast carcinoma samples. Only SVM-BT-RFE was able to partially predict the true biological nature of the data set. The feature enriched the term oncogenic signature (C6) “KRAS.600.LUNG.BREAST_UP.V1_UP” that comprised the genes that were up-regulated in epithelial lung and breast cancer cell lines overexpressing an oncogenic form of KRAS gene. The “sigFeature” and other given algorithms could not predict the biological nature of the data set. We needed further in-depth inquiry to define the appropriate reason why those algorithms were unable to detect the cancer signature.

Next, data set GSE25099 has the characteristic of oral squamous cell carcinoma. In this case, “sigFeature” and the other algorithms could not identify the biological signature, as they could not enrich any gene set containing features related to oral cancer. For further clarification, a differential expression analysis was performed between case and control samples, and a signature list was generated using a cutoff value of *p* < 0.05 and a fold change of ±1.5. This list of signatures was also unable to predict the cancer signature in the GSEA assessment.

Overall, the analysis revealed that “sigFeature” is able to predict the signature of three out of six data sets with absolute precision, whereas the fourth one partially (Table 2). Comparable proficiency was observed only with SVM-RFE (three of six). Support vector machine BT-RFE could get one out of six right and partially correct about the other, whereas SVM-T-RFE failed in case of all the data sets. Thus, it appears from the comparative analysis that selection of biologically relevant feature is a crucial achievement of the newly developed “sigFeature.”

**Table 2:** Results of GSEA.

	**Final score (out of 6)**	**GSE3744**	**GSE25099**	**GSE26712**	**GSE2280**	**GSE7670**	**GSE4290**
sigFeature	4	No	No	Yes	Yes	Yes	Yes (partially)
SVM-RFE	3	No	No	Yes	Yes	Yes	No
SVM-BT-RFE	2	Yes (partially)	No	No	No	Yes	No
SVM-T-RFE	1	No	No	No	No	No	No

**Figure 7:**
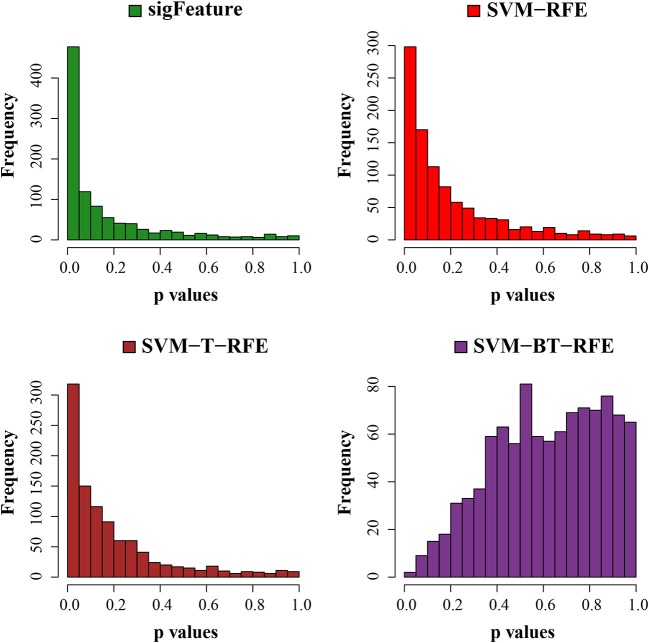
Histogram plots of unadjusted *p*-values (using top 1,000 robust features). The comparison of unadjusted *p*-values is shown, which are calculated by using the top 1,000 features (from the robust feature list) individually between the classes.

Next, we have tested the same *t*-test with robust list of features produced in each algorithm of selection. The *p*-values are graphically displayed below in Figure 7. The average unadjusted *p*-value generated by the top characteristics (*n* = 1,000) selected by the “sigFeature” algorithm is much more significant than the unadjusted *p*-value generated by the other selection algorithm. Also for the robust feature lists, the similar result is found.

## Discussion

In this article, we proposed a novel feature selection algorithm using “SVM-RFE” and *t* statistic (called “sigFeature”) to select significant features. We also tested the “sigFeature” algorithm on six publicly available microarray data sets, containing different biological attributes, and compared it with already existing similar type of algorithms (such as “SVM-RFE,” “SVM-T-RFE,” and “SVM-BT-RFE”). The plots show that the sigFeature algorithm's initial goal of selecting significant feature along with excellent classification accuracy is being met. The top features chosen by the sigFeature algorithm are very considerably distinct from those chosen by other feature selection algorithms used in this research work. Thus, the average *p*-value plot indicates significant results for the features chosen by our algorithm. We compared the *p*-value produced by the top 1,000 features using those algorithms; it shows that more significant *p*-value s are generated by the features that are selected by “sigFeature” algorithm than others.

We could also predict robust biomarkers with a concrete focus on microarray studies on six cancer diagnosis data sets using sigFeature algorithm. The stability of the markers is appropriate for both reproducibility and biological validation. However, stability alone is not a nice performance measure, as it is easy to improve stability by considering some fixed sets of features. Moreover, the ensuing predictive model is probably poor in the classification of new samples. The CLA technique is an experimental methodology for evaluating the strength of biomarker lists in combination with the predictive results of classification designs based on them. This CLA protocol repeatedly considers some samples for selecting markers as well as estimating classifiers from autonomous samples used to assess the efficiency of classification. The external 10-fold cross-validation error is more convenient to evaluate the predictive performance of data sets with unbalanced class proportions, a common situation for microarray experiments. “sigFeature” shows promising performance compared to other feature selection algorithms.

Finally, performing GSEA analysis using specified cancer gene sets of MSigDB, we obtained a concrete evidence to show that features selected by “sigFeature” have the potential to identify biological attributes of data sets more accurately. Here, “sigFeature” was able to predict the signature of three out of six data sets with complete accuracy, whereas the fourth one partially. Comparable expertise was observed with SVM-RFE, whereas the rest have put up a comparatively poor show.

Thus, we can conclude that the proposed algorithm identifies features leading to more accurate classification and generation of differentially significant features.

## Data Availability Statement

Publicly available datasets were analyzed in this study. This data can be found here: GSE3744, GSE25099, GSE26712, GSE2280, GSE7670, and GSE4290.

## Ethics Statement

Ethical review and approval was not required for the study on human participants in accordance with the local legislation and institutional requirements. The ethics committee waived the requirement of written informed consent for participation.

## Author Contributions

PD developed the method and implemented the sigFeature algorithms, under the supervision of ST and SR. All authors (PD, AR, SD, SR, and ST) added to composing the content, read and affirmed the final version of the manuscript.

### Conflict of Interest

The authors declare that the research was conducted in the absence of any commercial or financial relationships that could be construed as a potential conflict of interest.

## Supplementary Material

Supplementary Data Sheet 1 It contains overview of different feature selection algorithms used in this research work.

Supplementary Data Sheet 2 It contains experimental results of different data sets.

Supplementary Data Sheet 3 It contains experimental results on RNA-Seq data set.
