# Supplementary material for: sigFeature: Novel Significant Feature Selection Method for Classification of Gene Expression Data Using Support Vector Machine and t Statistic
Source: Front Genet. 2020 Apr 3;11:247. doi: 10.3389/fgene.2020.00247 (PMC7169426; doi:10.3389/fgene.2020.00247)
Supplement: Supplementary Data Sheet 1 — It contains overview of different feature selection algorithms used in this research work. [file Data_Sheet_1.docx]

**Supplementary Material: Overview of Different Feature Selection Algorithms**

Pijush Das^1,*^, Anirban Roychowdhury^2^, Subhadeep Das^1^, Susanta Roychoudhury^3^ and Sucheta Tripathy^1,4*^

^1^Computational Genomics lab, Structural Biology and Bioinformatics Division, CSIR- Indian Institute of Chemical Biology; Kolkata -700032, ^2^Department of Oncogene Regulation, Chittaranjan National Cancer Institute, 37, S.P. Mukherjee Road, Kolkata, West Bengal 700026, India, ^3^Saroj Gupta Cancer Centre and Research Institute, Mahatma Gandhi Road, Thakurpukur, Calcutta - 700063, INDIA. ^4^ Academy of Scientific and Innovative Research, New Delhi, India.

In this supplementary material, we summarize the main working principle of four different feature selection algorithms such as SVM-RFE, SVM-T-RFE and SVM-BT-RFE which are used in this analysis. All the feature selection algorithms are based on support vector machine (SVM) which are used for selecting features for binary classification. A detailed description of those algorithms is given in this section.

# 2.2.1 Support Vector Machine-Recursive Feature Elimination (SVM-RFE)

In 2002 Guyon, et al. ([Guyon, et al., 2002](#_ENREF_2)) introduced a feature selection method known as support vector machine recursive feature elimination (SVM-RFE) for classification of cancer. In the literature, it is accepted that the feature ranking coefficients can be used as classifier weights. The most informative features are those which correspond to the largest weight. Thus a sequential backward feature elimination procedure is used by SVM-RFE for selecting the feature with the smallest weight that is subsequently stored into a stack. This iteration process is continued until the last feature variable remains.

For calculating the feature ranking score at every progression, the weight vector$w$ of a linear SVM is used. The smallest ranking score of an *i*^th^ feature $c_{i}=(w_{i}^{2})$is eliminated where$w_{i}$represents the corresponding component in the weight vector$w$. This can also be elucidated as weighted sums of support vectors ([Zhang, et al., 2006](#_ENREF_6)).

For further illustration ([Zhou and Tuck, 2007](#_ENREF_7)), the squared coefficients $c_{i}=(w_{i}^{2})$ are used as the ranking criterion and the feature corresponding to the smallest value is removed. The removal of this feature changes the objective function (*J*) to the smallest extent (Where $i=1,...,p$are the slack variables). The magnitude of$w_{i}^{2}$ corresponds to the approximate change in the criterion shown in **Equation 2** when the *j*^th^ feature is repudiated.

|  | $J=\frac{1}{2}\vert\vert w\vert\vert^{2}+C\sum_{i=1}^{n} \xi_{i}$ | (2) |
| --- | --- | --- |

The equation (2) is described by the Optimal Brain Damage (OBD) algorithm, ([LeCun, et al., 1990](#_ENREF_3)). The equation (3) estimates the change in objective function originated by eliminating a given feature by expanding the objective function (*J*) using Taylor series to second order:

|  | $\Delta J(i)=\frac{\partial J}{\partial w_{i}}\Delta w_{i}+\frac{\partial^{2}J}{\partial w_{i}^{2}}(\Delta w_{i})^{2}+O((\Delta w_{i})^{3})$ | (3) |
| --- | --- | --- |

At the most appropriate of *J*, the first-order term can be disregarded, and equation (3) changes to:

|  | $\Delta J(i)\approx(\Delta w_{i})^{2}$ | (4) |
| --- | --- | --- |

Here *J(i)* is the value of *J* after the *i*^th^ feature is eliminated (by setting the corresponding weight to 0), the equation changes into:

|  | $J(i)\approx J+w_{i}^{2}$ | (5) |
| --- | --- | --- |

Therefore, eliminating the feature which has the smallest$w_{i}^{2}$will cause the slightest increase in *J* thereby increasing the generalisation performance. In other words, “SVM-RFE” focuses to find the feature subset that is dependent on negligible criterion *J.* We have observed that “SVM-RFE” fails to identify differentially significant features that are essential for classification. The ranking procedure used in “SVM-RFE” is inadequate to find the DEGs and hence an alternative modified method is required.

# 2.2.2 SVM-T-RFE

Another extended version of the “SVM-RFE” algorithm was introduced by ([Li, et al., 2012](#_ENREF_4)) named as “SVM-T-RFE” for recognizing metastasis-related features (genes) in colorectal cancer using gene expression profiles. In this algorithm, two different methods e.g.; statistical t-test, and “SVM-RFE” algorithm is incorporated. The statistical t-test is well known for identifying the differentially expressed features (genes) between two samples in gene expression data. In this algorithm, two sample Welch's t-test is conducted which is calculated as given below.

|  | $t=\frac{\bar{x}_{1}-\bar{x}_{2}}{\sqrt{\frac{s_{1}^{2}}{n_{1}}+\frac{s_{2}^{2}}{n_{2}}}}$ | (6) |
| --- | --- | --- |

Where the size of sample 1 and sample 2 are $n_{1}$ and $n_{2}$, the means of the sample1 and sample 2 are $\bar{x}_{1}$and$\bar{x}_{2}$ and the variance of sample1 and sample 2 are$s_{1}^{2}$and $s_{2}^{2}$ respectively. In this algorithm, the ranking score of *i*^th^ feature is calculated as follows:

|  | $r_{i}=\theta*w_{i}+(1-\theta)*t_{i}$ | (7) |
| --- | --- | --- |

Where$\theta$is the parameter influencing the tradeoff between SVM weight value and t-statistic. The features are ranked on the basis of the ranking score and the algorithm is followed backward feature elimination procedure like “SVM-RFE” algorithm. The value of $\theta$ lies between 0 and 1. Estimation of $\theta$ is a very cumbersome procedure (not discussed here) and needs big datasets possibly leading to better feature selection.

# 2.2.3 SVM-BT-RFE

The “SVM-BT-RFE” is another feature selection algorithm that is very similar to “SVM-T-RFE” ([Mishra and Mishra, 2015](#_ENREF_5)). The main goal behind the development of this algorithm is to achieve distinct biological facts by mining relevant genes. Instead of the statistical t-test, Bayesian T-test is used in this algorithm. The topmost features that are differentially significant with a p-value of ≤ 0.05 are used as the ranking criterion. The equation is given as below

|  | $Rank,R=T_{g}[\eta*W_{i}+(1-\eta)*B_{ti}]$ | (8) |
| --- | --- | --- |

Where $T_{g}$= p-value of topmost features produced from the Bayesian T-test;$\eta$= parametric concurrence between SVM weight and Bayesian T-test score; $W_{i}$= SVM weight vector for the *i*^th^ feature and $B_{ti}$= Bayesian T-test value (p-value) for all *i*^th^ features. In this algorithm, the features are ranked on basis of ranking value and the algorithm follows backward elimination procedure like “SVM-RFE” algorithm. The value of $\eta$ lies between 0 and 1. Determination of$\eta$ is again an extremely tedious procedure.

**References**

Furey, T.S.*, et al.* (2000) Support vector machine classification and validation of cancer tissue samples using microarray expression data, *Bioinformatics*, **16**, 906-914.

Guyon, I.*, et al.* (2002) Gene selection for cancer classification using support vector machines, *Machine learning*, **46**, 389-422.

LeCun, Y.*, et al.* (1990) Handwritten digit recognition with a back-propagation network. *Advances in neural information processing systems*. pp. 396-404.

Li, X.*, et al.* (2012) SVM–T-RFE: A novel gene selection algorithm for identifying metastasis-related genes in colorectal cancer using gene expression profiles, *Biochemical and biophysical research communications*, **419**, 148-153.

Mishra, S. and Mishra, D. (2015) SVM-BT-RFE: An improved gene selection framework using Bayesian T-test embedded in support vector machine (recursive feature elimination) algorithm, *Karbala International Journal of Modern Science*, **1**, 86-96.

Zhou, X. and Tuck, D.P. (2007) MSVM-RFE: extensions of SVM-RFE for multiclass gene selection on DNA microarray data, *Bioinformatics*, **23**, 1106-1114.
