# Supplementary material for: sigFeature: Novel Significant Feature Selection Method for Classification of Gene Expression Data Using Support Vector Machine and t Statistic
Source: Front Genet. 2020 Apr 3;11:247. doi: 10.3389/fgene.2020.00247 (PMC7169426; doi:10.3389/fgene.2020.00247)
Supplement: Supplementary Data Sheet 2 — It contains experimental results of different data sets. [file Data_Sheet_2.docx]

**Supplementary Material: Experimental Studies**

Pijush Das^1,*^, Anirban Roychowdhury^2^, Subhadeep Das^1^, Susanta Roychoudhury^3^ and Sucheta Tripathy^1,4*^

^1^Computational Genomics lab, Structural Biology and Bioinformatics Division, CSIR- Indian Institute of Chemical Biology; Kolkata -700032, ^2^Department of Oncogene Regulation, Chittaranjan National Cancer Institute, 37, S.P. Mukherjee Road, Kolkata, West Bengal 700026, India, ^3^Saroj Gupta Cancer Centre and Research Institute, Mahatma Gandhi Road, Thakurpukur, Calcutta - 700063, INDIA. ^4^ Academy of Scientific and Innovative Research, New Delhi, India.

In this supplementary material, we report the comparative data analysis results of the proposed “sigFeature” algorithm and three existing feature selection algorithms ( e.g. “SVM-RFE”, “SVM-T-RFE” and “SVM-BT-RFE”) on six publicly available different types of cancer microarray data sets such as: squamous cell carcinoma (GSE2280, Feb 19, 2005) (The results are included in the main article), breast cancer (GSE3744, Feb 09, 2006), brain cancer (GSE4290, Apr 10, 2006), lung cancer (GSE7670, Aug 30, 2007), oral cancer (GSE25099, Sep 01, 2011), ovarian cancer (GSE26712, Jan 20, 2011).

**Experimental Results**

**2. GSE3744 dataset**

**2.1. Bootstrap plot of the sample set**


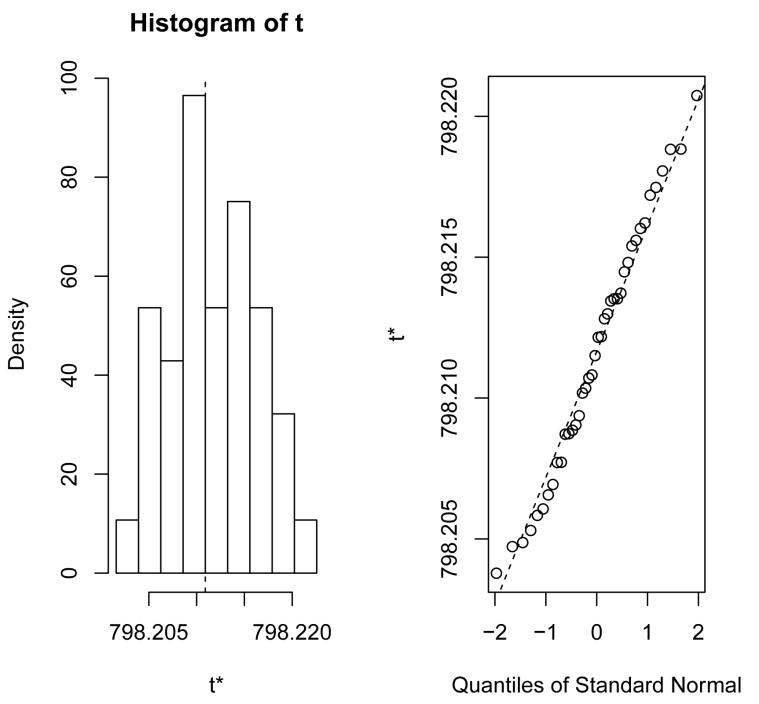


**Figure 2.1: Bootstrap distribution plot and Normal Q-Q plot for the dataset GSE3744.** We used 40 bootstraps (with replacement) with 90% of the total samples (GSE3744) to randomize the array of sub-samples.

**2.2. Stability of the selected features**


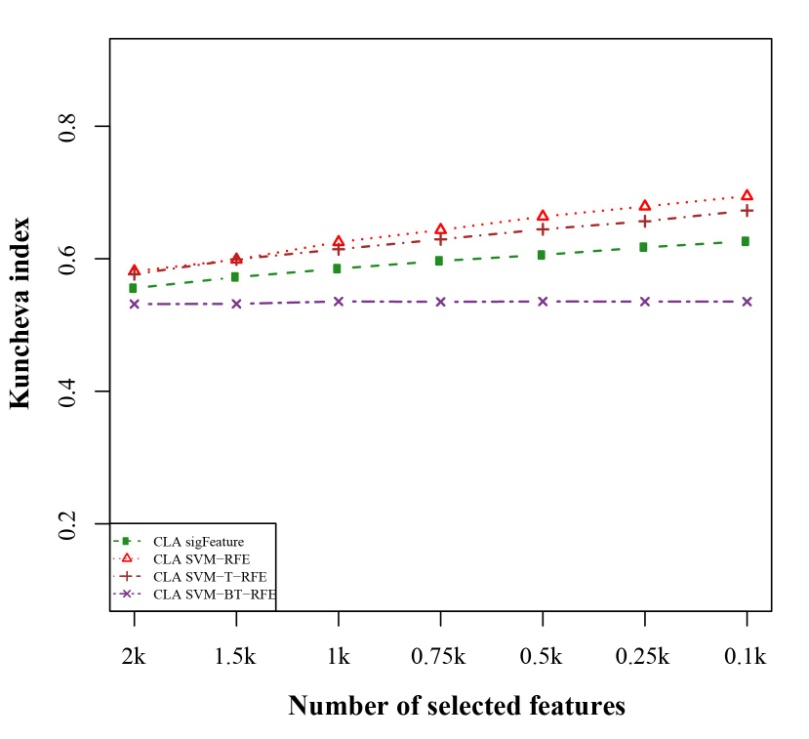


**Figure 2.2.1: Kuncheva index plot for the data set GSE3744.** The stability of the features are measured based on complete linear aggregation methods for different feature selection algorithms. We used 40 bootstraps (with replacement) and eliminated E=1% features.


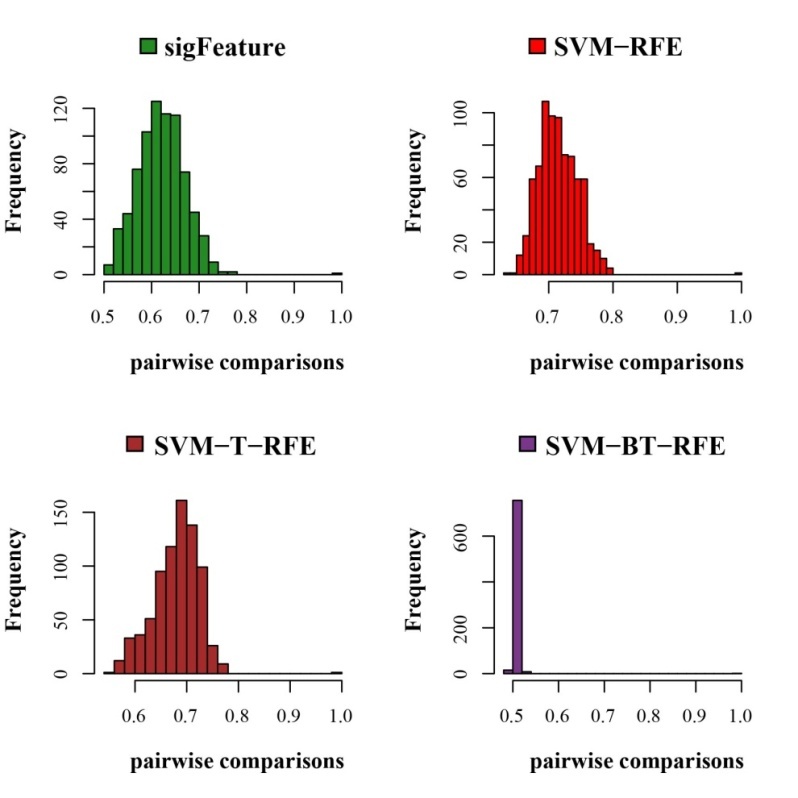


**Figure 2.2.2:** **Histogram plots for pair wise stability comparison of the features**. Distribution plots of the pairwise stabilities for the data set GSE3744 where different algorithms produce the feature lists. In each iteration of the algorithms, we used 40 bootstraps, eliminated E = 1% features, used a signature size of 10% and selected the CLA aggregation model.

**2.3. Classification performance result**

**
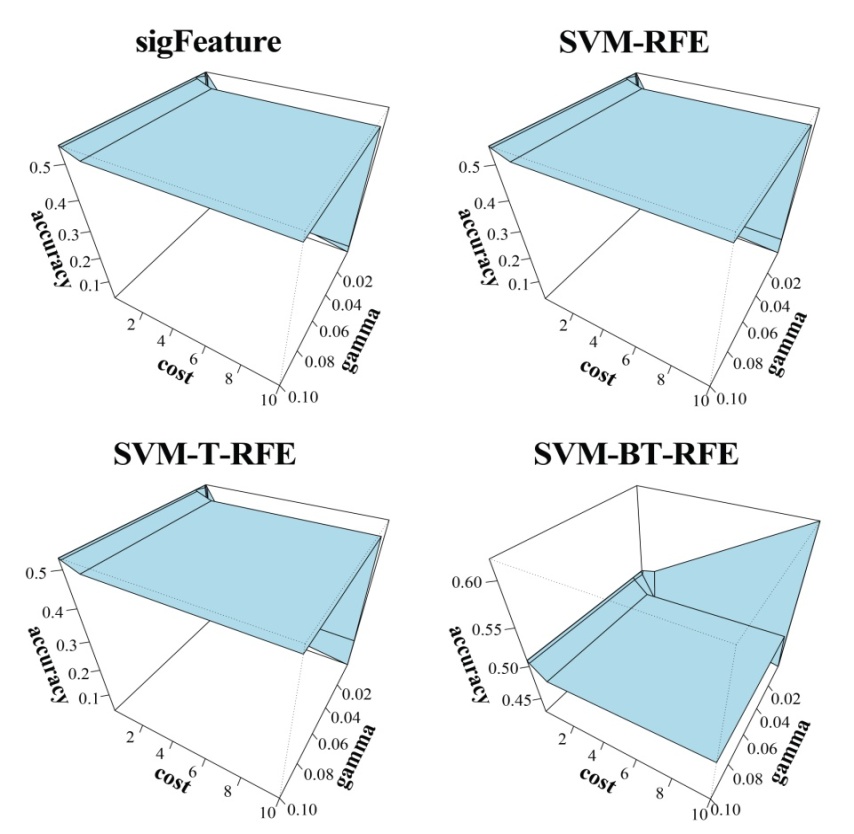
**

**Figure 2.3.1: 3D representation of cost, gamma and classification accuracy.** The Cost and gamma values are selected to determine the best performance in the classification of features selected (top one thousand features by CLA) by different selection algorithms.


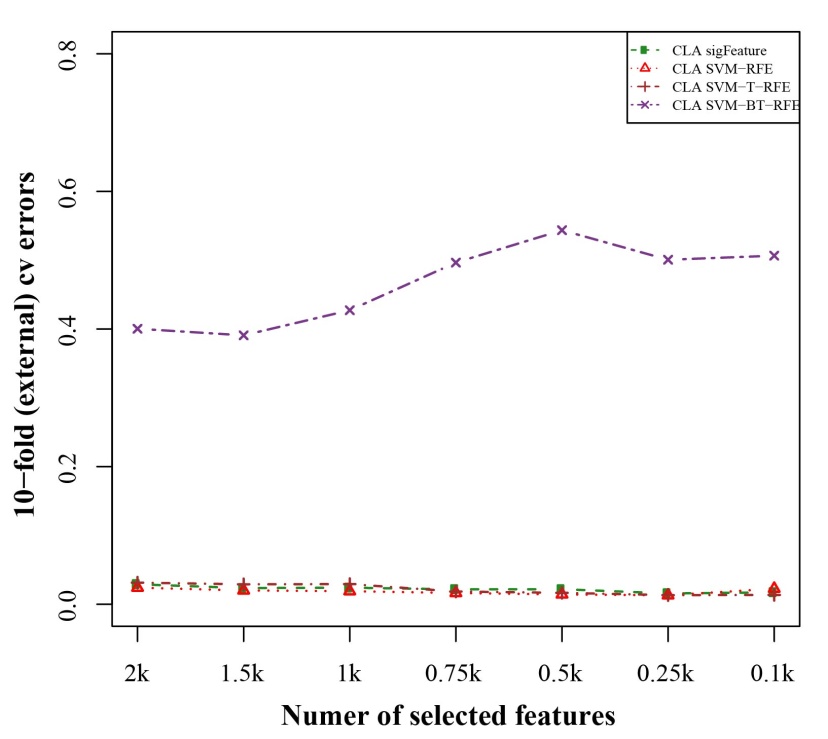


**Figure 2.3.2: 10-fold external cross-validation error plot.** The classification performances of the top features are shown here which are selected by different feature selection algorithms. We used 40 bootstraps and eliminated E=1% features.

**2.4. Differentially significant features**

**
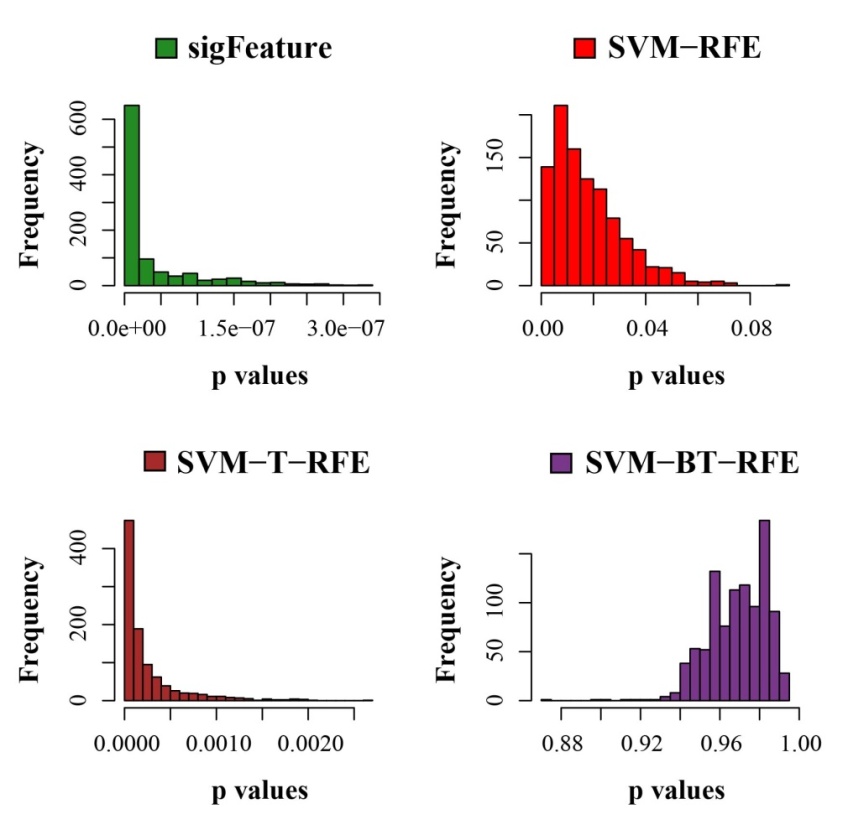
**

**Figure 2.4.1:** **Histogram plots of unadjusted p-values.** The comparison of the average unadjusted p-values are shown, which are calculated individually using the top one thousand features between classes. The list of features is made using 40 bootstrap sub-sets where the feature selection algorithms remove E = 1% features at each iteration.


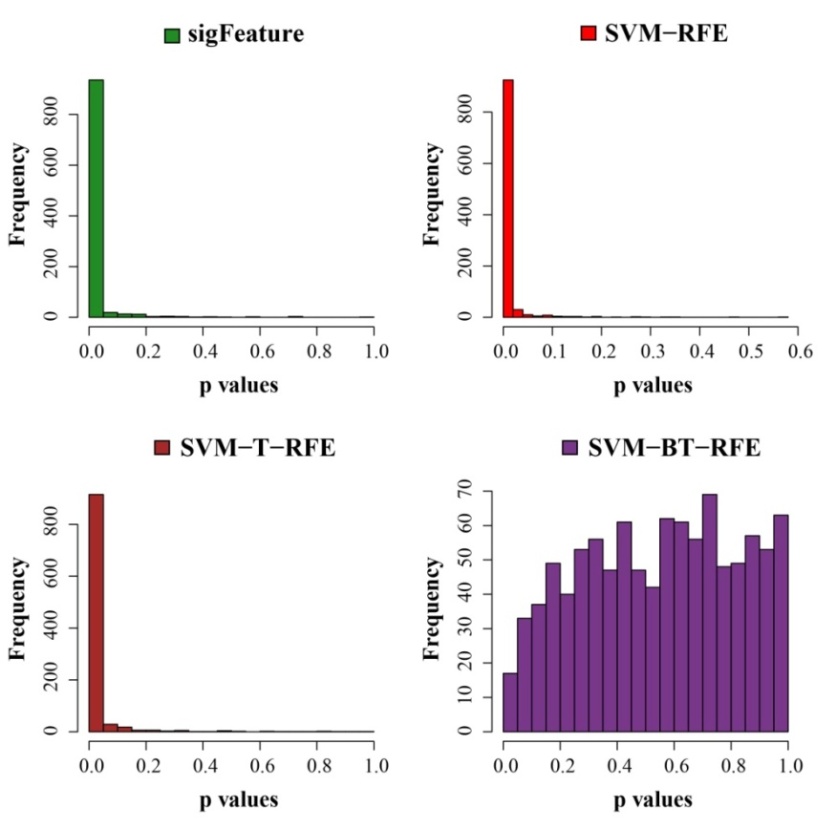


**Figure 2.4.2:** **Histogram plots of unadjusted p-values (using top 1000 robust features).** The comparison of unadjusted p-values are shown, which are calculated by using top one thousand features (from the robust feature list) individually between the classes.

**3. GSE4290 dataset**

**3.1. Bootstrap plot of the sample set**

**
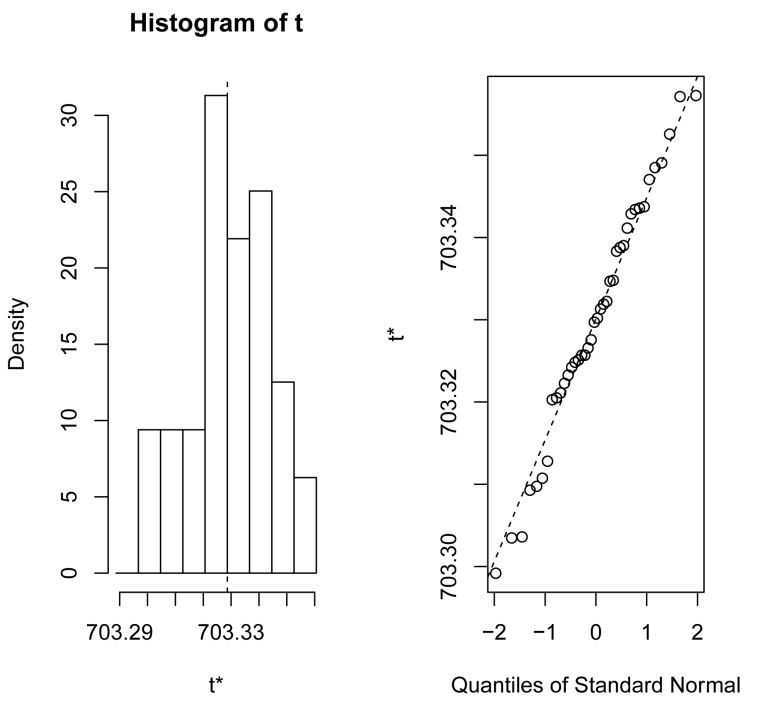
**

**Figure 3.1: Bootstrap distribution plot and Normal Q-Q plot for the dataset GSE4290.** We used 40 bootstraps (with replacement) with 90% of the total samples (GSE4290) to randomize the array of sub-samples

**3.2. Stability of the selected features**

**
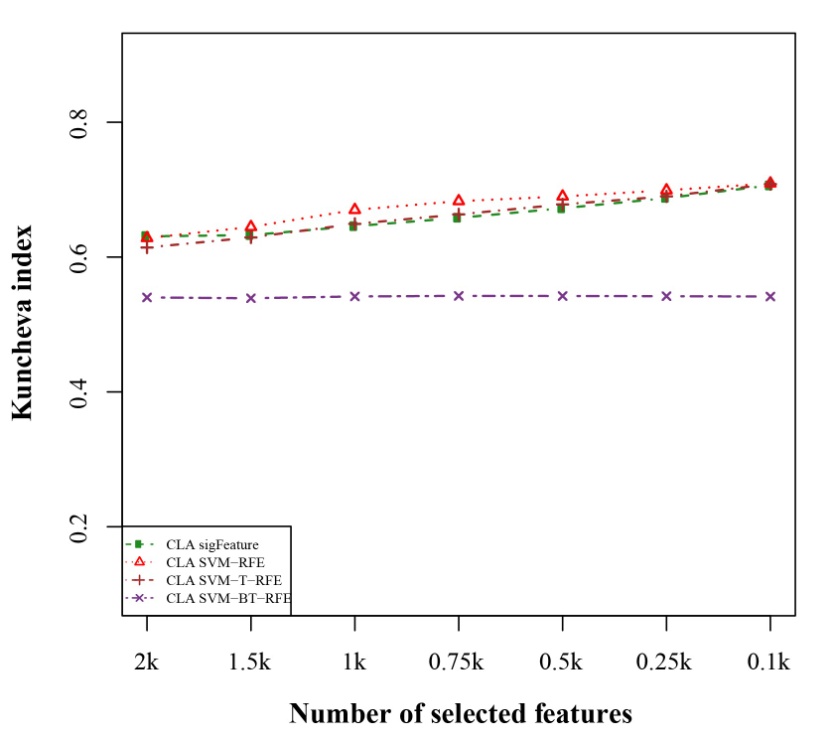
**

**Figure 3.2.1: Kuncheva index plot for the data set GSE4290.** The stability of the features are measured based on complete linear aggregation methods for different feature selection algorithms. We used 40 bootstraps (with replacement) and eliminated E=1% features.

**
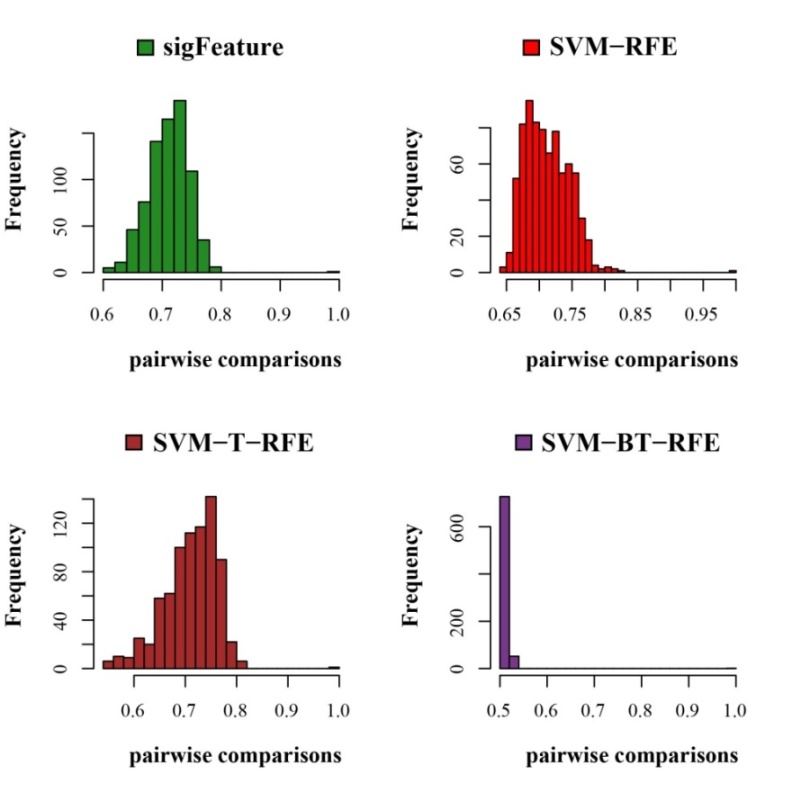
**

**Figure 3.2.2:** **Histogram plots for pair wise stability comparison of the features.** Distribution plots of the pairwise stabilities for the data set GSE4290 where different algorithms produce the feature lists. In each iteration of the algorithms, we used 40 bootstraps, eliminated E = 1% features, used a signature size of 10% and selected the CLA aggregation model.

**3.3. Classification performance result**


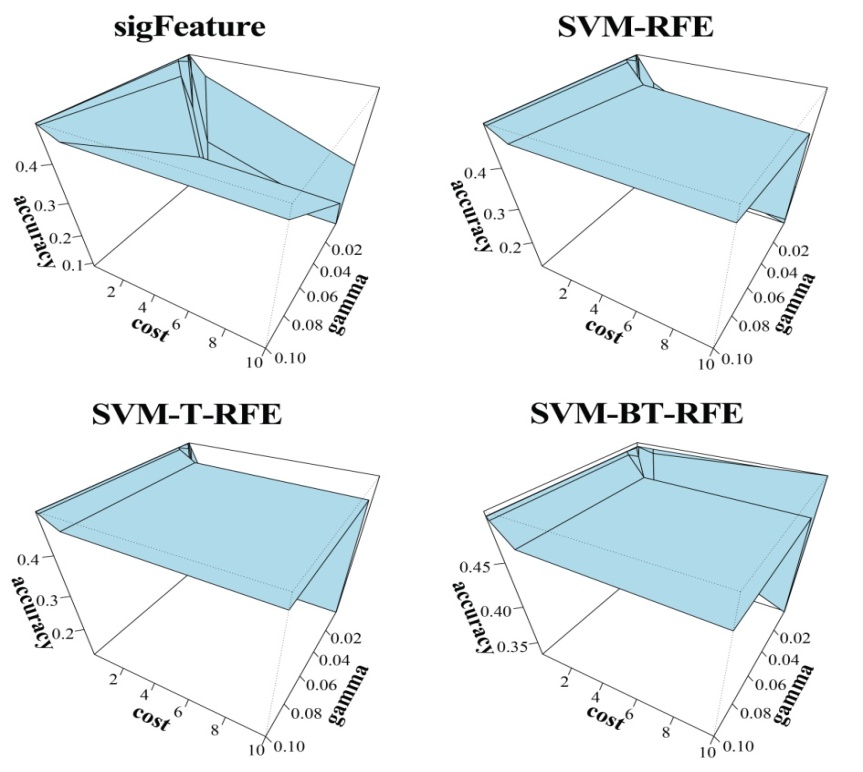


**Figure 3.3.1: 3D representation of cost, gamma and classification accuracy.** The Cost and gamma values are selected to determine the best performance in the classification of features selected (top one thousand features by CLA) by different selection algorithms.


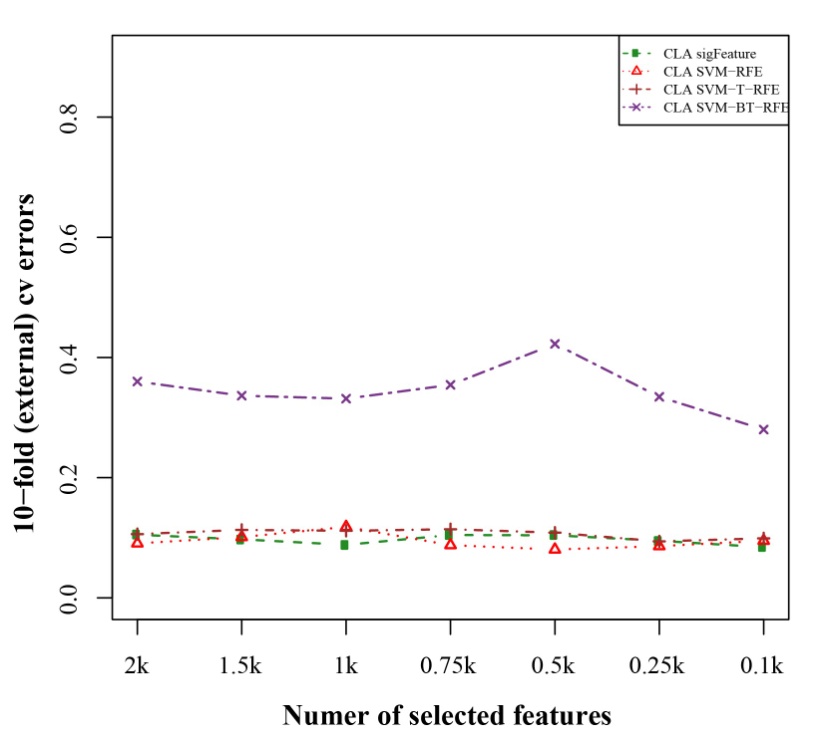


**Figure 3.3.2: 10-fold external cross-validation error plot.** The classification performances of the top features are shown here which are selected by different feature selection algorithms. We used 40 bootstraps and eliminated E=1% features.

**3.4. Differentially significant features**


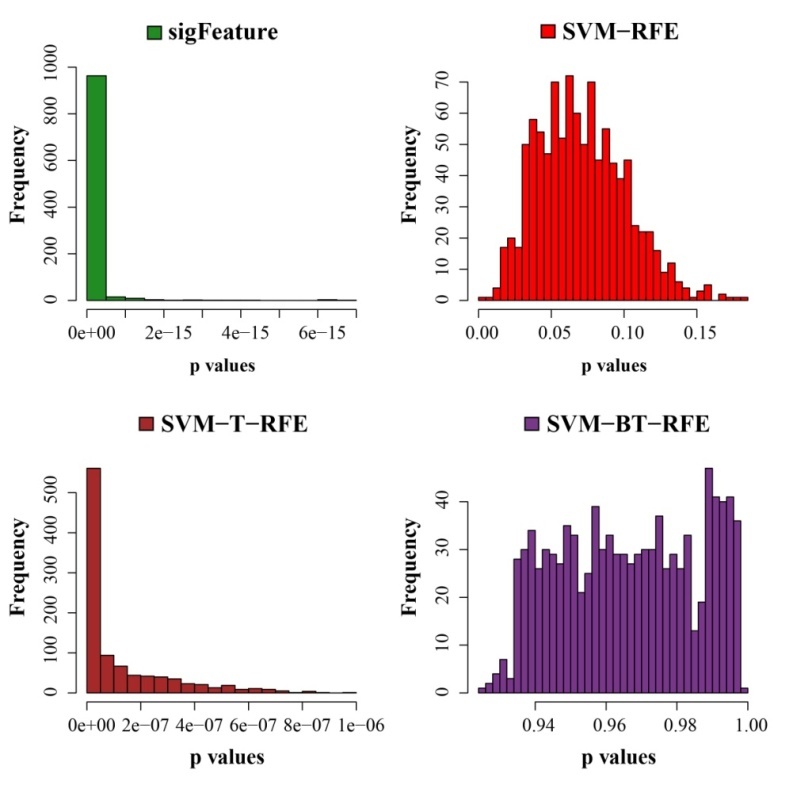


**Figure 3.4.1:** **Histogram plots of unadjusted p-values**. The comparison of the average unadjusted p-values are shown, which are calculated individually using the top one thousand features between classes. The list of features is made using 40 bootstrap sub-sets where the feature selection algorithms remove E = 1% features at each iteration.


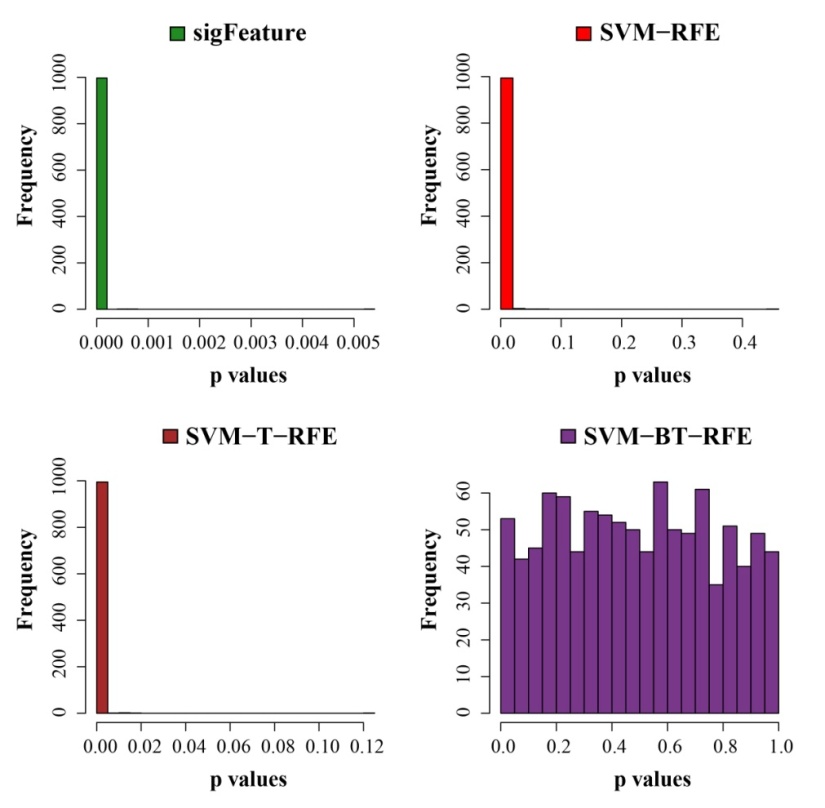


**Figure 3.4.2:** **Histogram plots of unadjusted p-values (using top 1000 robust features).** The comparison of unadjusted p-values are shown, which are calculated by using top one thousand features (from the robust feature list) individually between the classes.

**4. GSE7670 dataset**

**4.1. Bootstrap plot of the sample set**


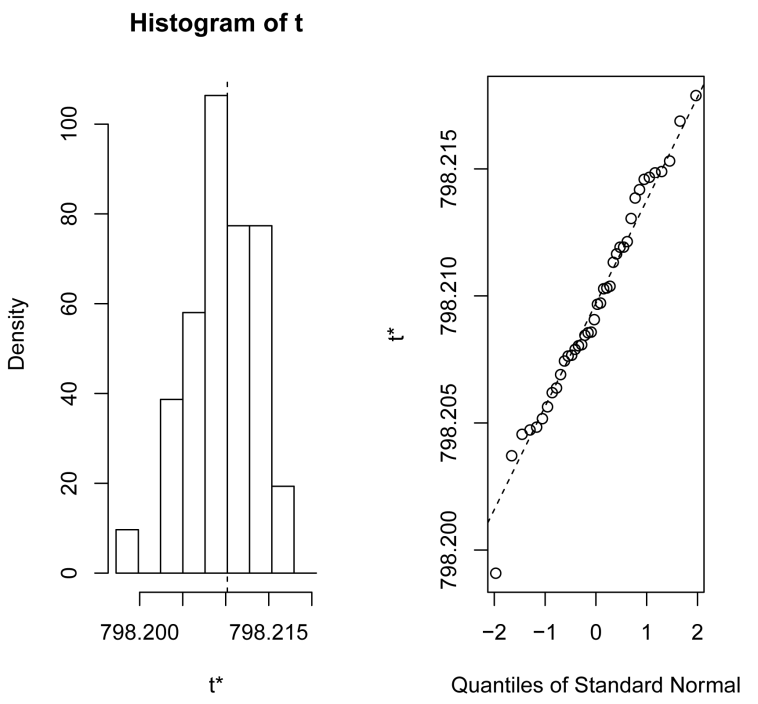


**Figure 4.1: Bootstrap distribution plot and Normal Q-Q plot for the dataset GSE7670.** We used 40 bootstraps (with replacement) with 90% of the total samples (GSE7670) to randomize the array of sub-samples.

**4.2. Stability of the selected features**


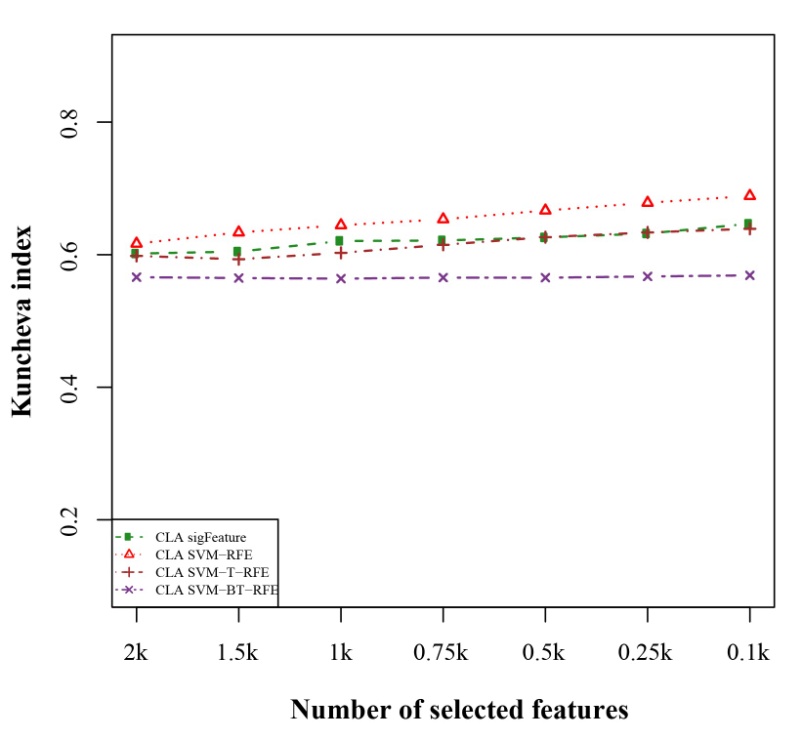


**Figure 4.2.1: Kuncheva index plot for the data set GSE7670.** The stability of the features are measured based on complete linear aggregation methods for different feature selection algorithms. We used 40 bootstraps (with replacement) and eliminated E=1% features.


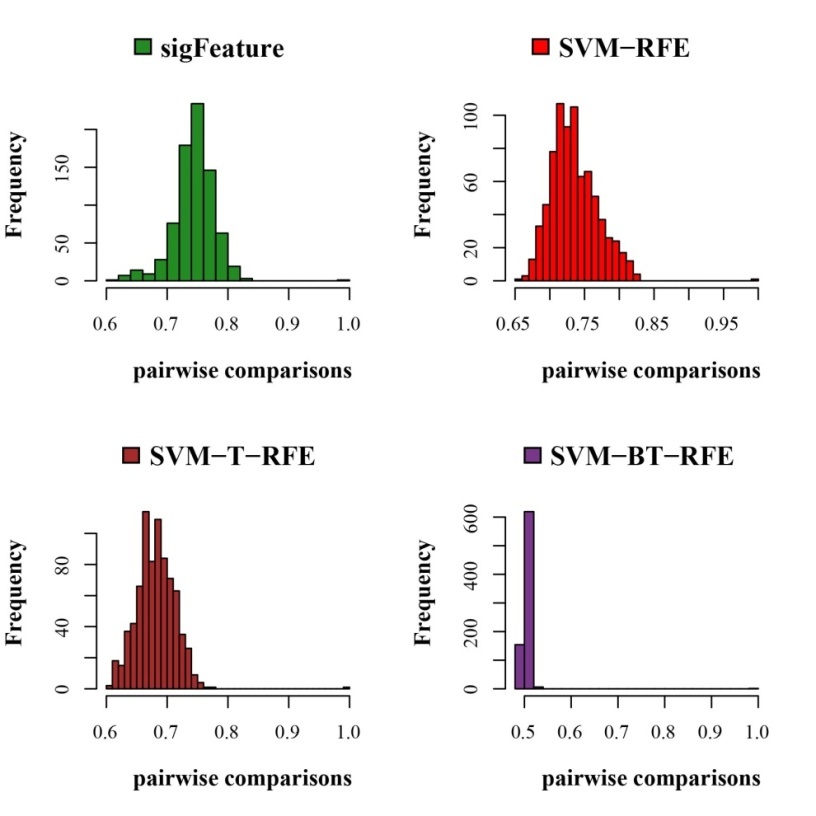


**Figure 4.2.2:** **Histogram plots for pair wise stability comparison of the features**. Distribution plots of the pairwise stabilities for the data set GSE7670 where different algorithms produce the feature lists. In each iteration of the algorithms, we used 40 bootstraps, eliminated E = 1% features, used a signature size of 10% and selected the CLA aggregation model.

**4.3. Classification performance result**


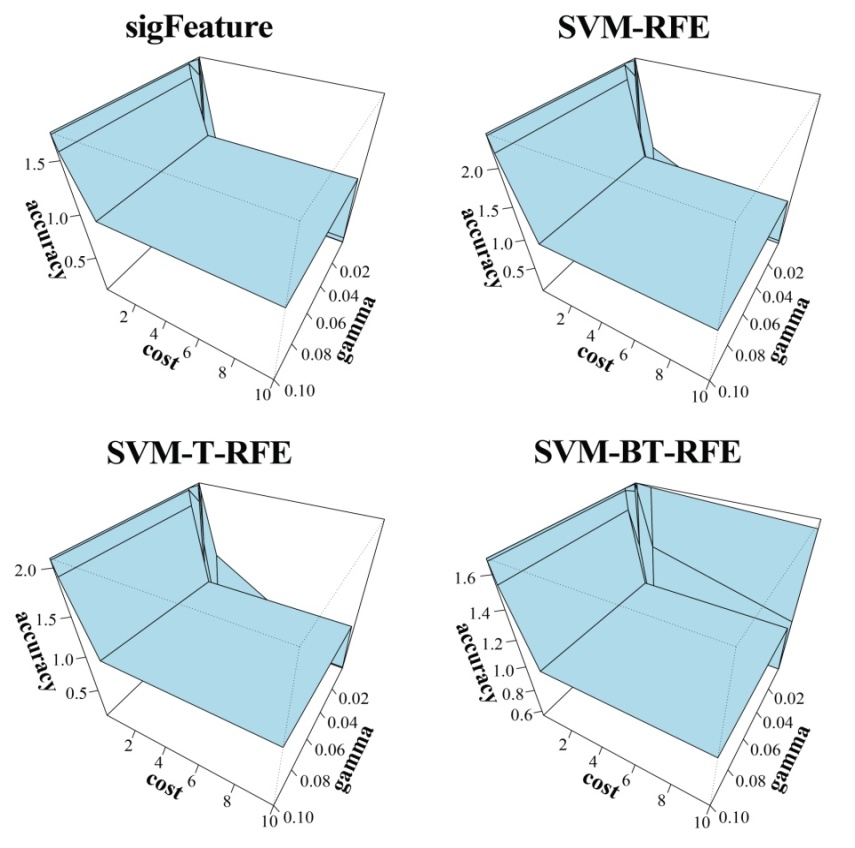


**Figure 4.3.1: 3D representation of cost, gamma and classification accuracy.** The Cost and gamma values are selected to determine the best performance in the classification of features selected (top one thousand features by CLA) by different selection algorithms.


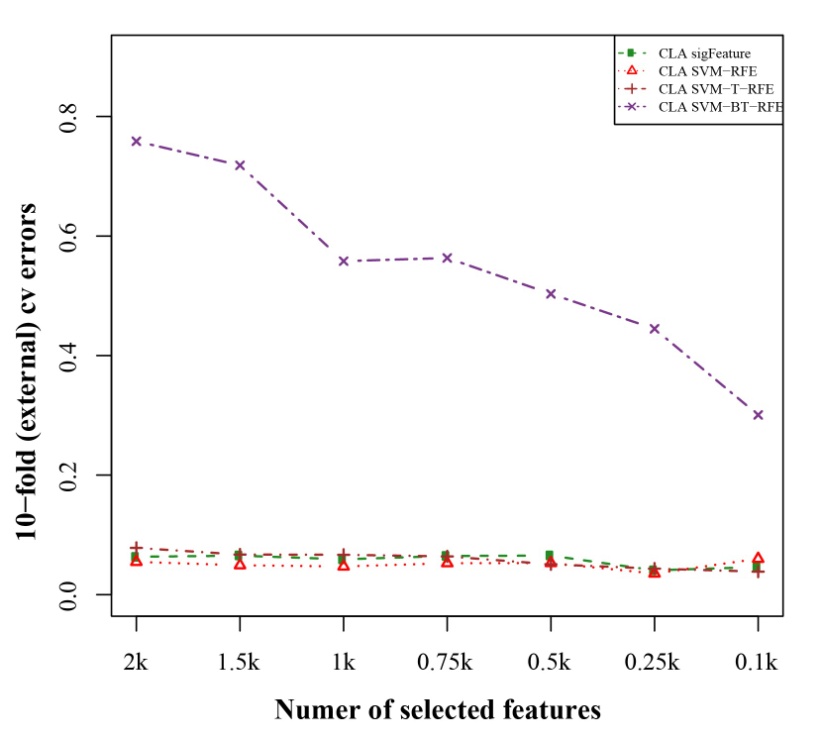


**Figure 4.3.2: 10-fold external cross-validation error plot**. The classification performances of the top features are shown here which are selected by different feature selection algorithms. We used 40 bootstraps and eliminated E=1% features.

**4.4. Differentially significant features**


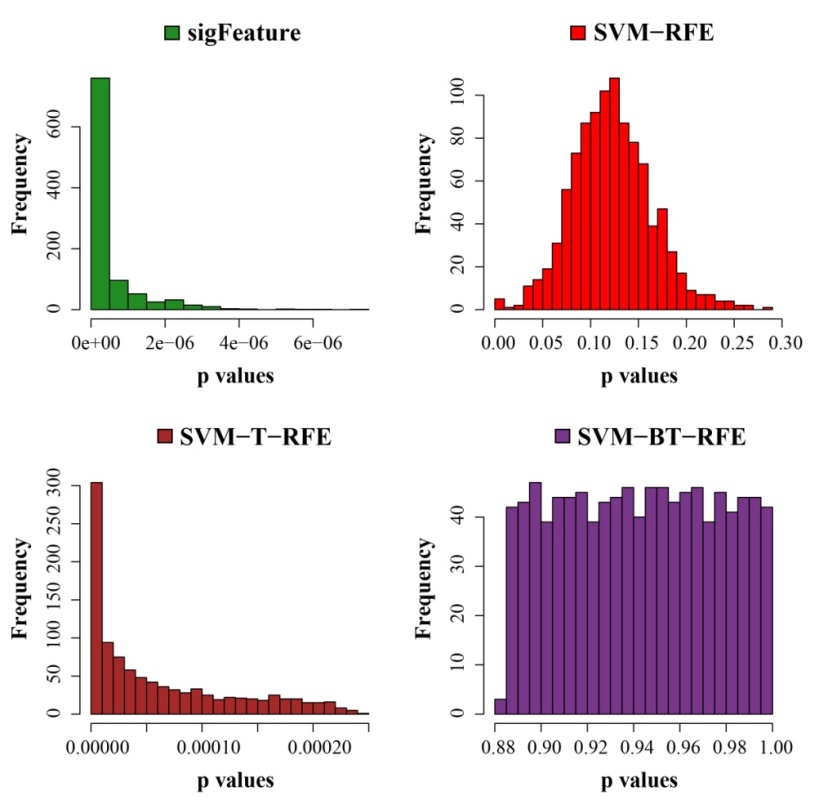


**Figure 4.4.1:** **Histogram plots of unadjusted p-values.** The comparison of the average unadjusted p-values are shown, which are calculated individually using the top one thousand features between classes. The list of features is made using 40 bootstrap sub-sets where the feature selection algorithms remove E = 1% features at each iteration.


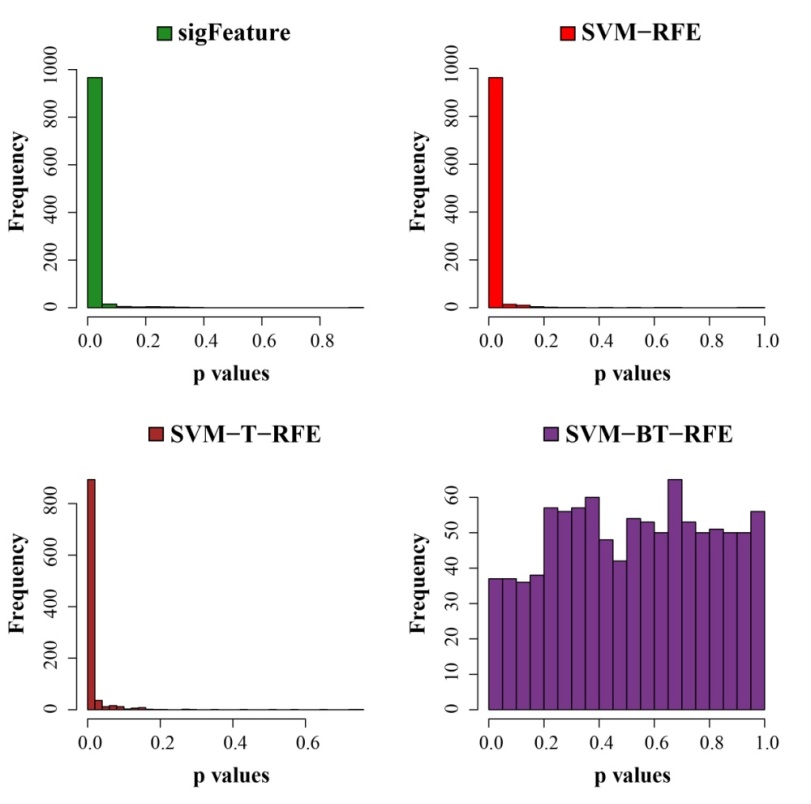


**Figure 4.4.2:** **Histogram plots of unadjusted p-values (using top 1000 robust features).** The comparison of unadjusted p-values are shown, which are calculated by using top one thousand features (from the robust feature list) individually between the classes.

**5. GSE25099 dataset**

**5.1. Bootstrap plot of the sample set**

**
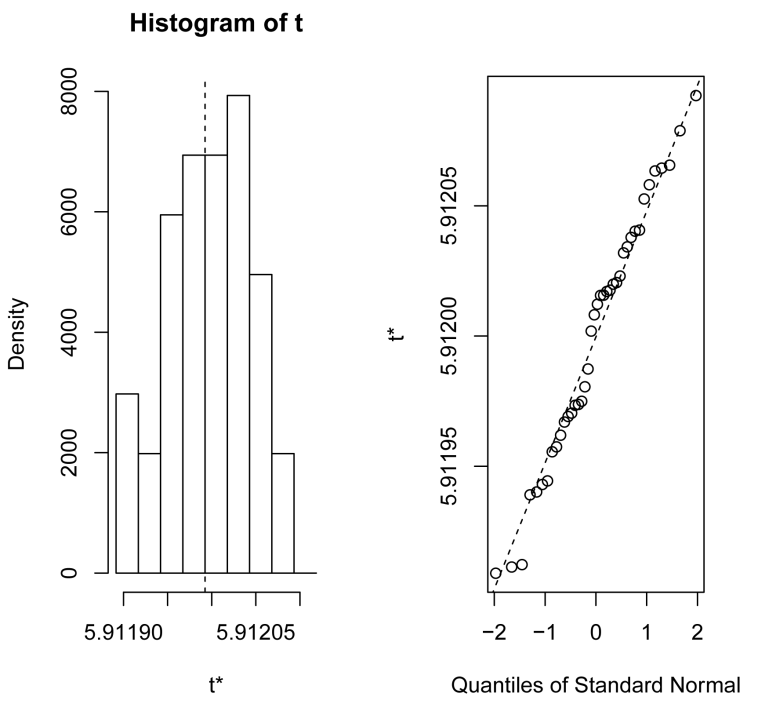
**

**Figure 5.1: Bootstrap distribution plot and Normal Q-Q plot for the dataset GSE25099.** We used 40 bootstraps (with replacement) with 90% of the total samples (GSE25099) to randomize the array of sub-samples.

**5.2. Stability of the selected features**

**
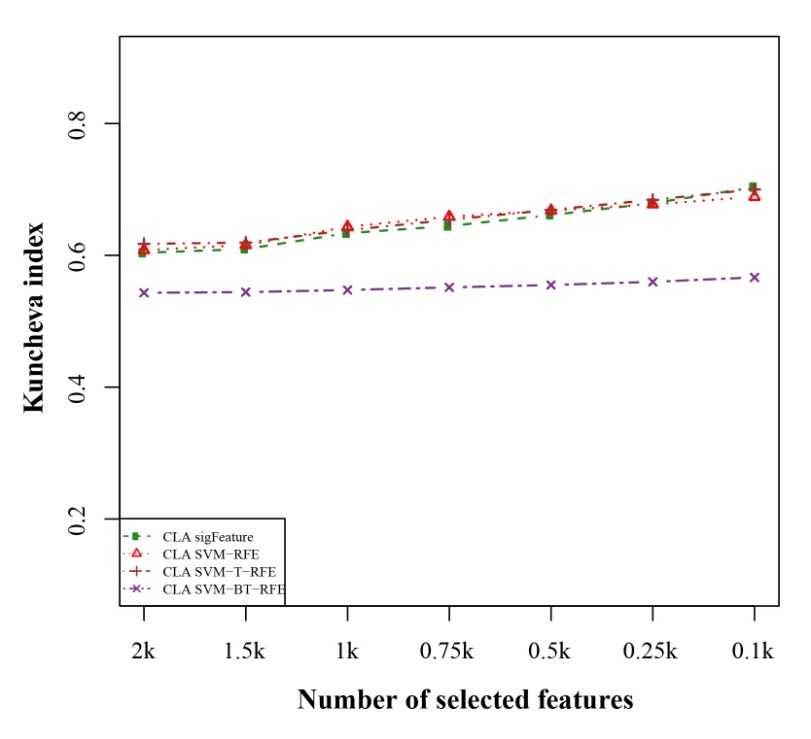
**

**Figure 5.2.1: Kuncheva index plot for the data set GSE25099.** The stability of the features are measured based on complete linear aggregation methods for different feature selection algorithms. We used 40 bootstraps (with replacement) and eliminated E=1% features.


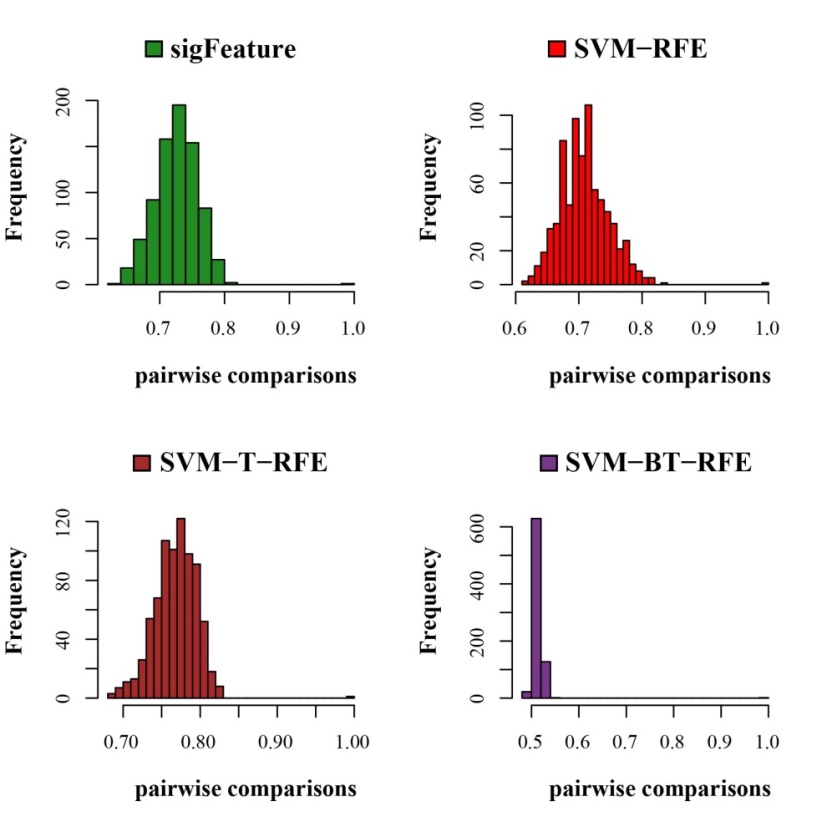


**Figure 5.2.2:** **Histogram plots for pair wise stability comparison of the features**. Distribution plots of the pairwise stabilities for the data set GSE25099 where different algorithms produce the feature lists. In each iteration of the algorithms, we used 40 bootstraps, eliminated E = 1% features, used a signature size of 10% and selected the CLA aggregation model.

**5.3. Classification performance result**


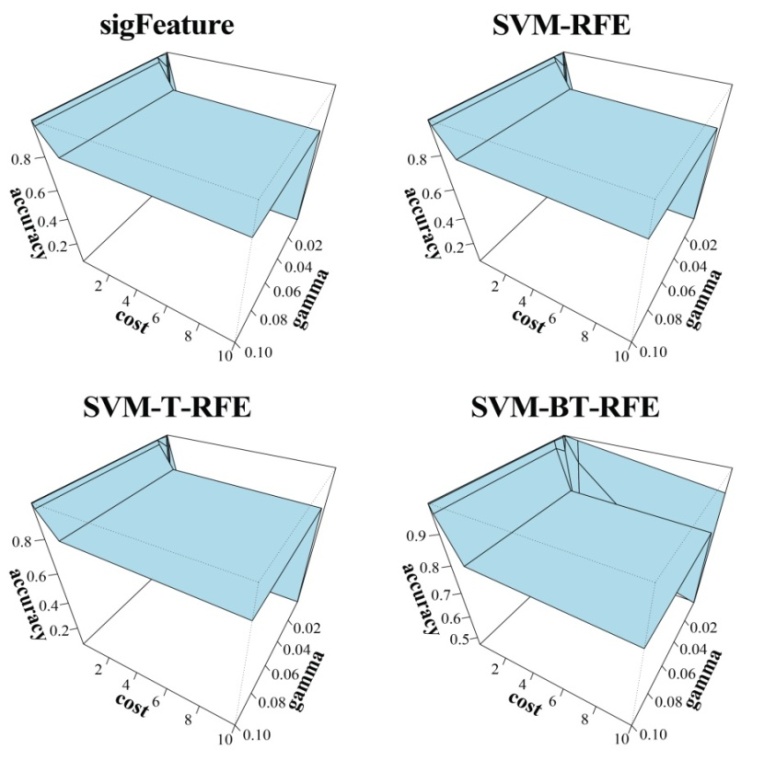


**Figure 5.3.1: 3D representation of cost, gamma and classification accuracy.** The Cost and gamma values are selected to determine the best performance in the classification of features selected (top one thousand features by CLA) by different selection algorithms.


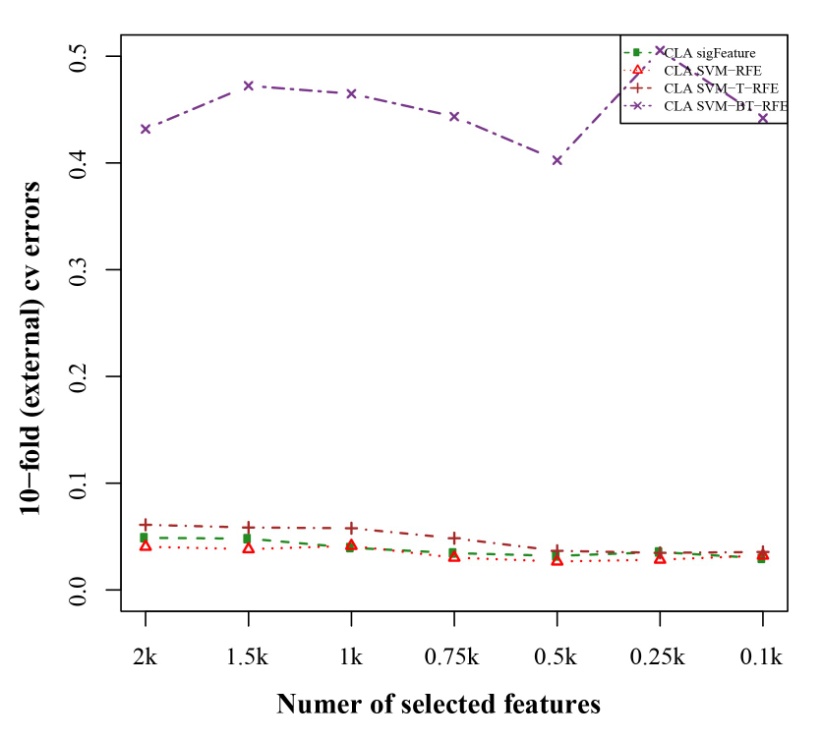


**Figure 5.3.2: 10-fold external cross-validation error plot.** The classification performances of the top features are shown here which are selected by different feature selection algorithms. We used 40 bootstraps and eliminated E=1% features.

**5.4. Differentially significant features**


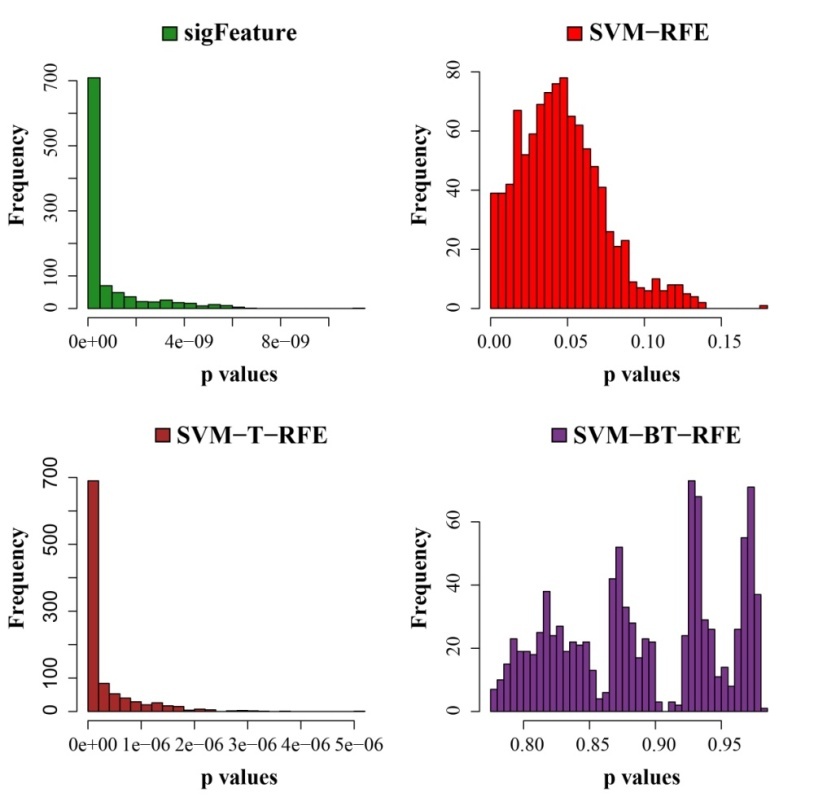


**Figure 5.4.1:** **Histogram plots of unadjusted p-values.** The comparison of the average unadjusted p-values are shown, which are calculated individually using the top one thousand features between classes. The list of features is made using 40 bootstrap sub-sets where the feature selection algorithms remove E = 1% features at each iteration.


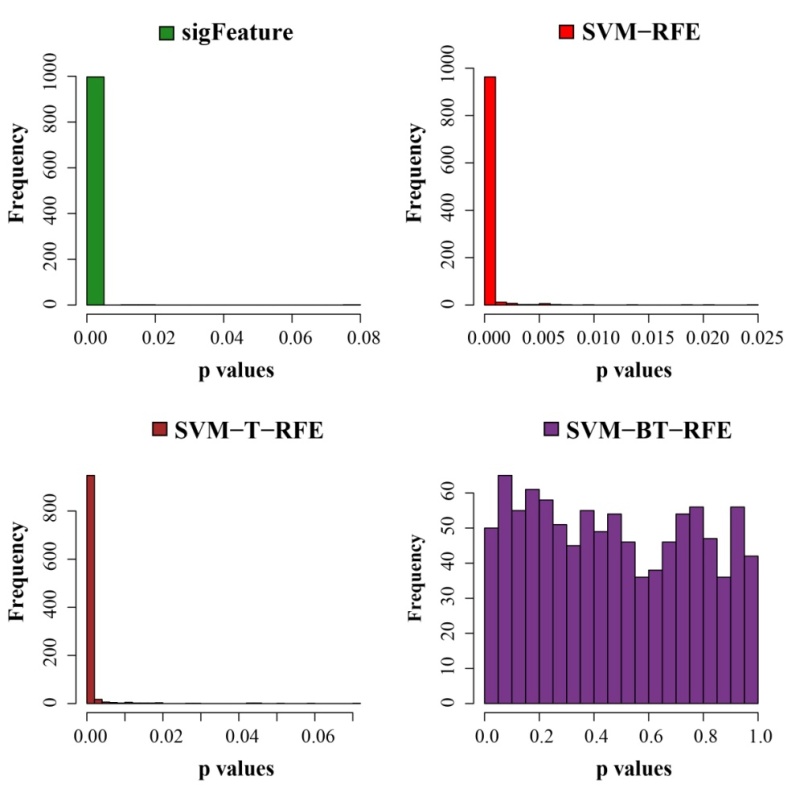


**Figure 5.4.2:** **Histogram plots of unadjusted p-values (using top 1000 robust features).** The comparison of unadjusted p-values are shown, which are calculated by using top one thousand features (from the robust feature list) individually between the classes.

**6. GSE26712 dataset**

**6.1. Bootstrap plot of the sample set**

**
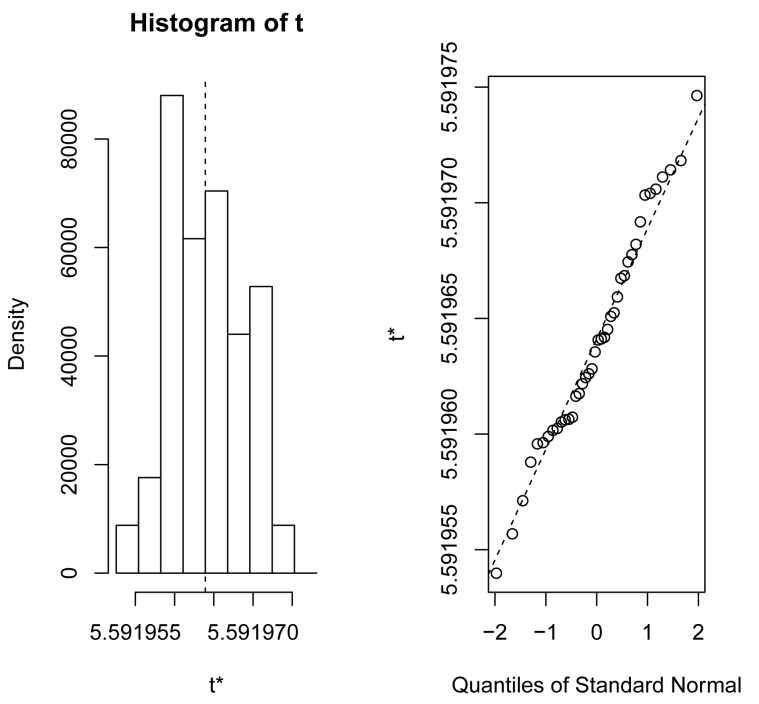
**

**Figure 6.1: Bootstrap distribution plot and Normal Q-Q plot for the dataset GSE26712.** We used 40 bootstraps (with replacement) with 90% of the total samples (GSE26712) to randomize the array of sub-samples.

**6.2. Stability of the selected features**


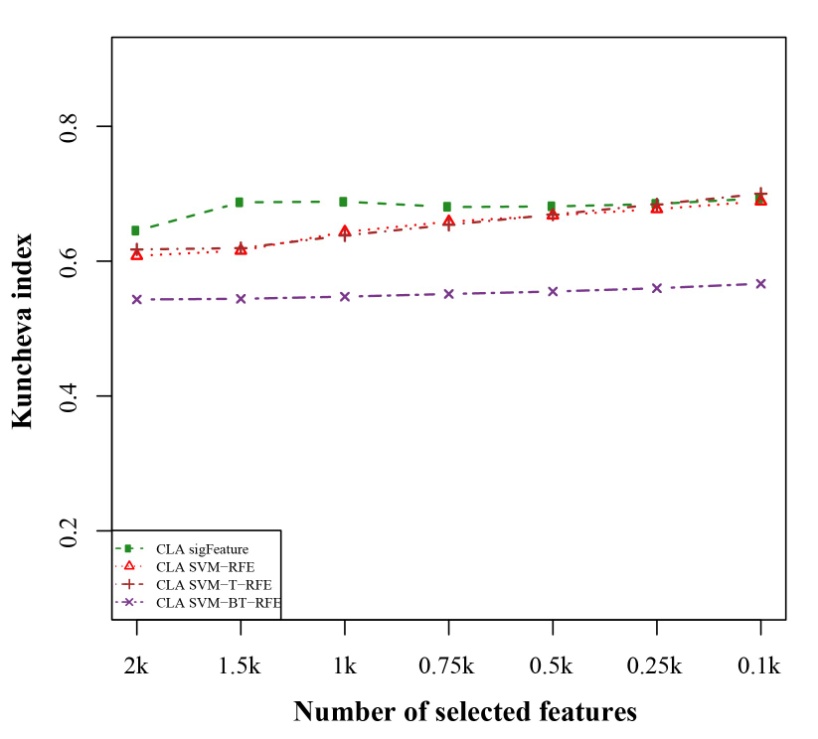


**Figure 6.2.1: Kuncheva index plot for the data set GSE26712.** The stability of the features are measured based on complete linear aggregation methods for different feature selection algorithms. We used 40 bootstraps (with replacement) and eliminated E=1% features.


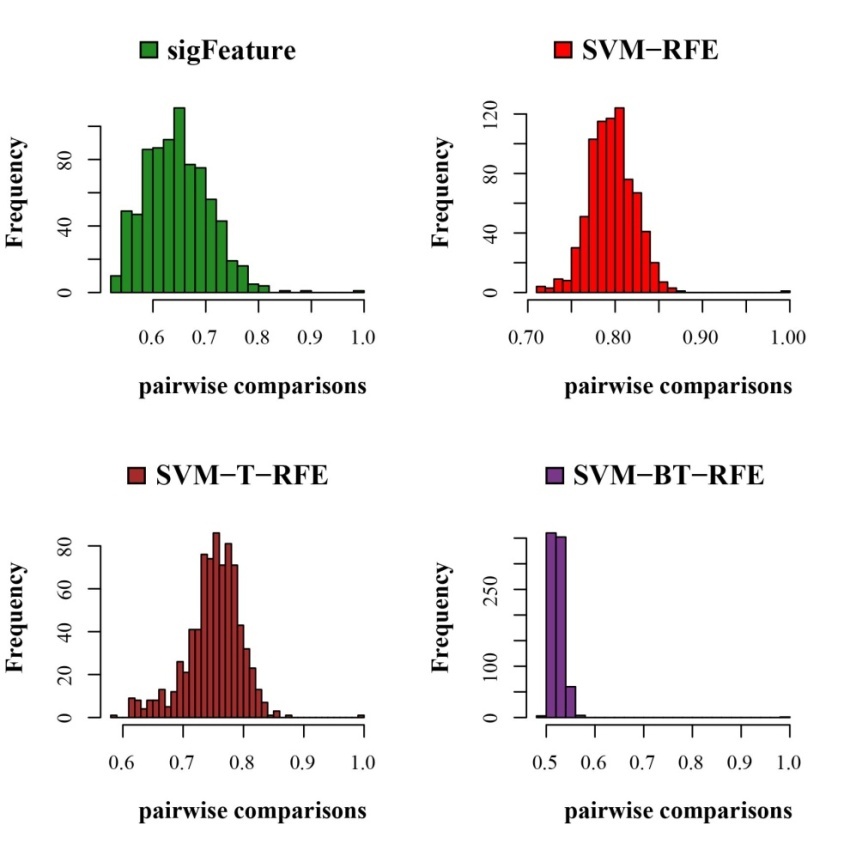


**Figure 6.2.2:** **Histogram plots for pair wise stability comparison of the features**. Distribution plots of the pairwise stabilities for the data set GSE26712 where different algorithms produce the feature lists. In each iteration of the algorithms, we used 40 bootstraps, eliminated E = 1% features, used a signature size of 10% and selected the CLA aggregation model.

**6.3. Classification performance result**


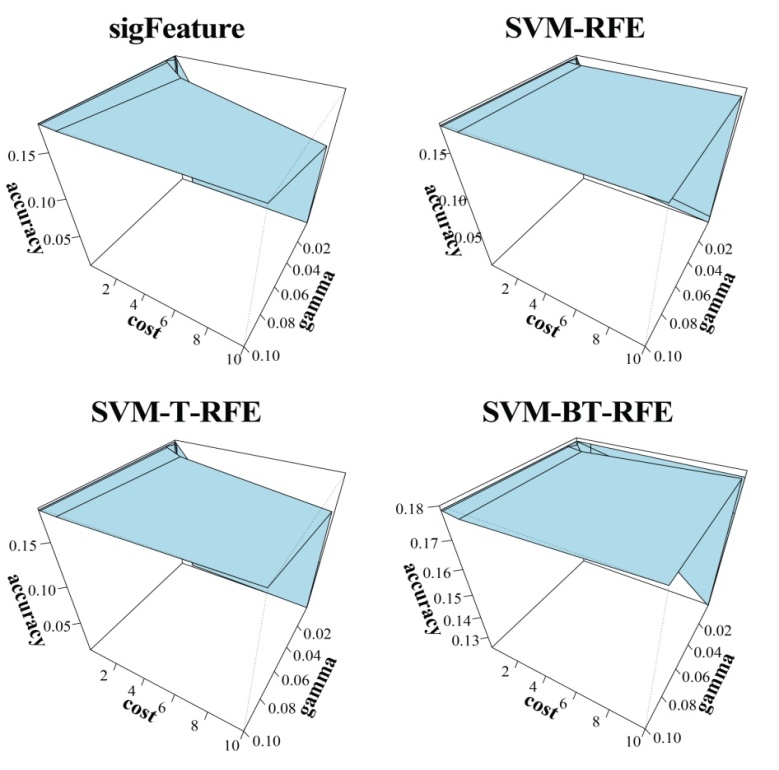


**Figure 6.3.1: 3D representation of cost, gamma and classification accuracy.** The Cost and gamma values are selected to determine the best performance in the classification of features selected (top one thousand features by CLA) by different selection algorithms.


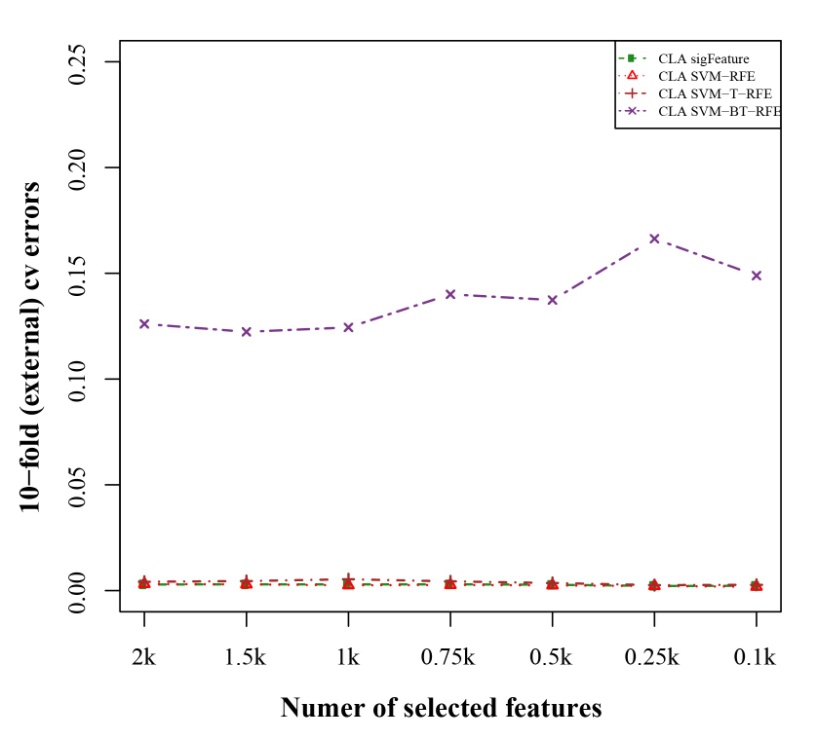


**Figure 6.3.2: 10-fold external cross-validation error plot.** The classification performances of the top features are shown here which are selected by different feature selection algorithms. We used 40 bootstraps and eliminated E=1% features.

**6.4. Differentially significant features**

**
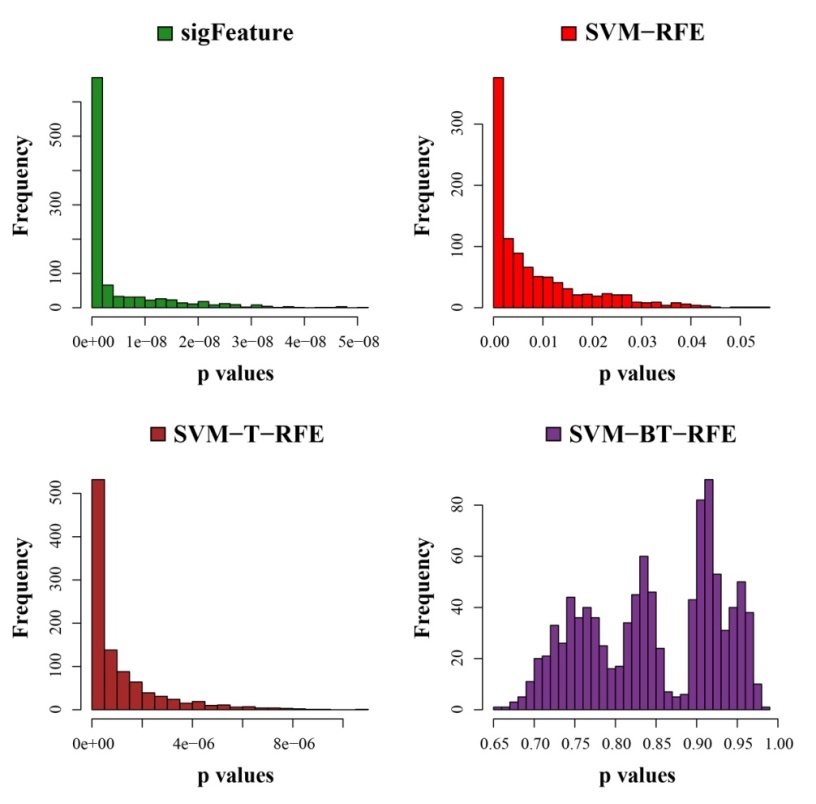
**

**Figure 6.4.1:** **Histogram plots of unadjusted p-values.** The comparison of the average unadjusted p-values are shown, which are calculated individually using the top one thousand features between classes. The list of features is made using 40 bootstrap sub-sets where the feature selection algorithms remove E = 1% features at each iteration.


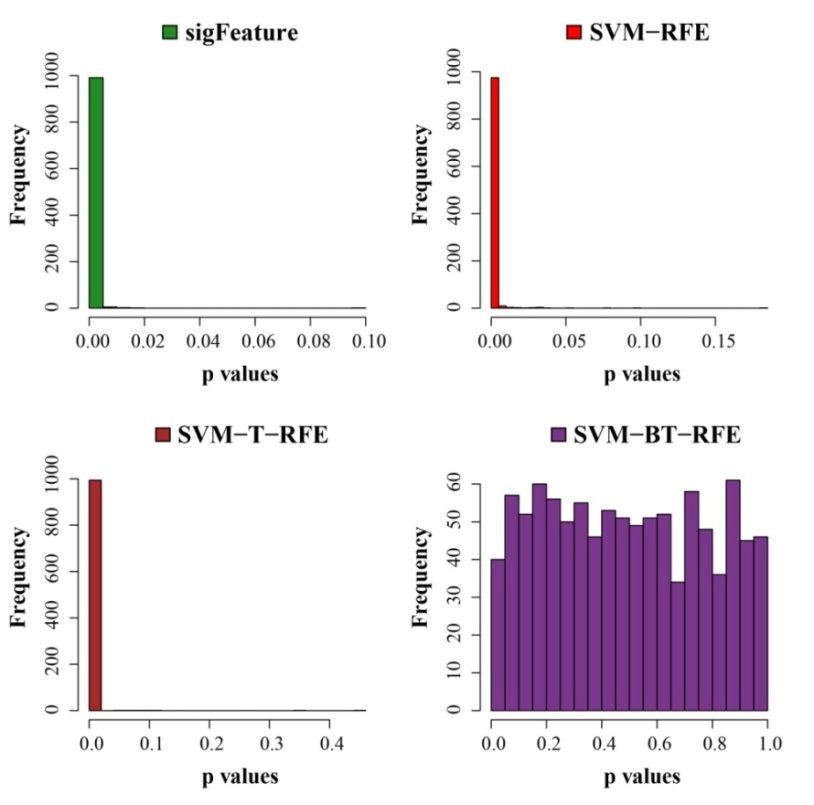


**Figure 6.4.2:** **Histogram plots of unadjusted p-values (using top 1000 robust features).** The comparison of unadjusted p-values are shown, which are calculated by using top one thousand features (from the robust feature list) individually between the classes.
