# Supplementary material for: sigFeature: Novel Significant Feature Selection Method for Classification of Gene Expression Data Using Support Vector Machine and t Statistic
Source: Front Genet. 2020 Apr 3;11:247. doi: 10.3389/fgene.2020.00247 (PMC7169426; doi:10.3389/fgene.2020.00247)
Supplement: Supplementary Data Sheet 3 — It contains experimental results on RNA-Seq data set. [file Data_Sheet_3.docx]

**Supplementary Material: Experimental Studies**

Pijush Das^1,*^, Anirban Roychowdhury^2^, Subhadeep Das^1^, Susanta Roychoudhury^3^ and Sucheta Tripathy^1,4*^

^1^Computational Genomics lab, Structural Biology and Bioinformatics Division, CSIR- Indian Institute of Chemical Biology; Kolkata -700032, ^2^Department of Oncogene Regulation, Chittaranjan National Cancer Institute, 37, S.P. Mukherjee Road, Kolkata, West Bengal 700026, India, ^3^Saroj Gupta Cancer Centre and Research Institute, Mahatma Gandhi Road, Thakurpukur, Calcutta - 700063, INDIA. ^4^ Academy of Scientific and Innovative Research, New Delhi, India.

In this supplementary material, we report the comparative data analysis results of the proposed “sigFeature” algorithm and three existing feature selection algorithms (e.g. “SVM-RFE”, “SVM-T-RFE” and “SVM-BT-RFE”) on a publicly available RNA-Seq data set (GSE119810). The overall perfomance and accuracy of feature selection was found to be similar as with other cancer microarray datasets.

**Experimental Results on RNA-Seq data set.**

**2. GSE119810 data set**

A crucial part of RNA-Seq data analysis using several feature selection algorithms such as “sigFeature”, “SVM-RFE”, “SVM-T-RFE” and “SVM-BT-RFE” is data normalization. It can be specified as determining and correcting the systematic variations to allow the analysis of samples in the same scale. Such systematic variations can result from both sample variations, including library size (sequencing depth) and the presence of majority fragments; and variations within the sample, including gene length and sequence composition (GC content).

We have used a Bioconductor package called “MLSeq” which contains two effective normalization methods for RNA-Seq data normalization. The first is the “deseq median ratio normalization”, which estimates the size factors by dividing each sample by geometric means of the transcript counts. The other method is “trimmed mean of M values (TMM)”. To minimize the log-fold differences between the samples and by absolute amplitude (default 5%), TMM first trims the data on both the lower and upper sides by log-fold changes (default 30%). TMM calculates a normalization factor after trimming, using the weighted mean of data. Using the delta method, these weights are calculated on the basis of inverse approximate asymptotic variances.

In this RNA-Seq data (GSE119810) analysis we have used “deseq median ratio normalization” for normalizing the data set. The remaining part of the feature selection procedure is the same as described in the main article.

**2.1. Bootstrap plot of the sample set**

**
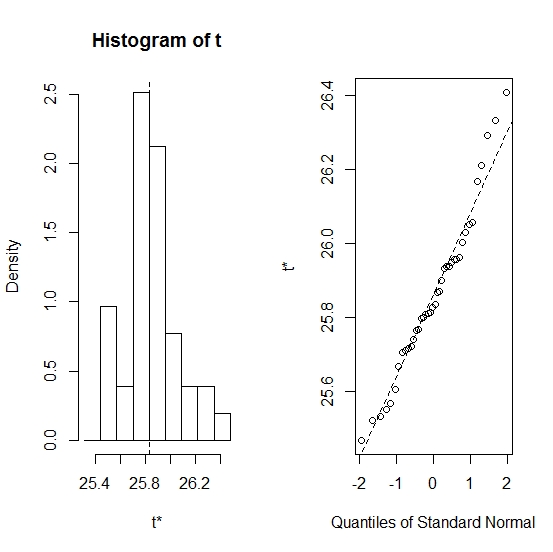
**

**Figure 1: Bootstrap distribution plot and Normal Q-Q plot for the dataset GSE119810.** We used 40 bootstraps (with replacement) with 90% of the total samples (GSE119810) to randomize the array of sub-samples.

**2.2. Stability of the selected features**

**
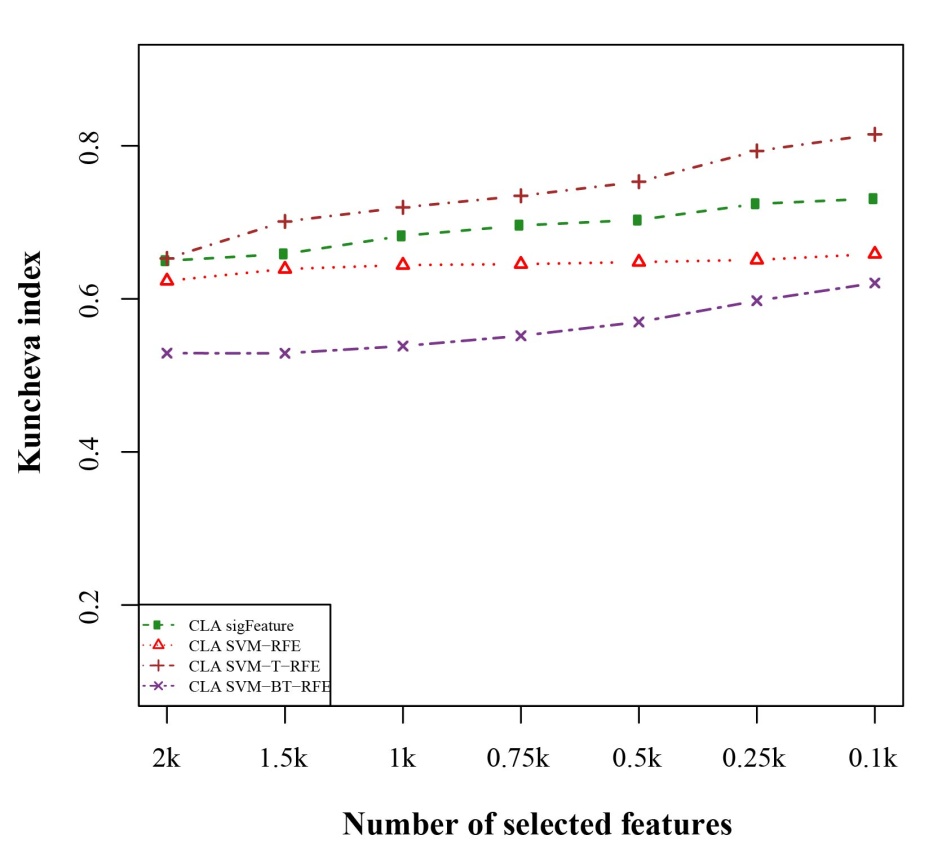
**

**Figure 2: Kuncheva index plot for the data set GSE119810.** The stability of the features are measured based on complete linear aggregation methods for different feature selection algorithms. We used 40 bootstraps (with replacement) and eliminated E=1% features.


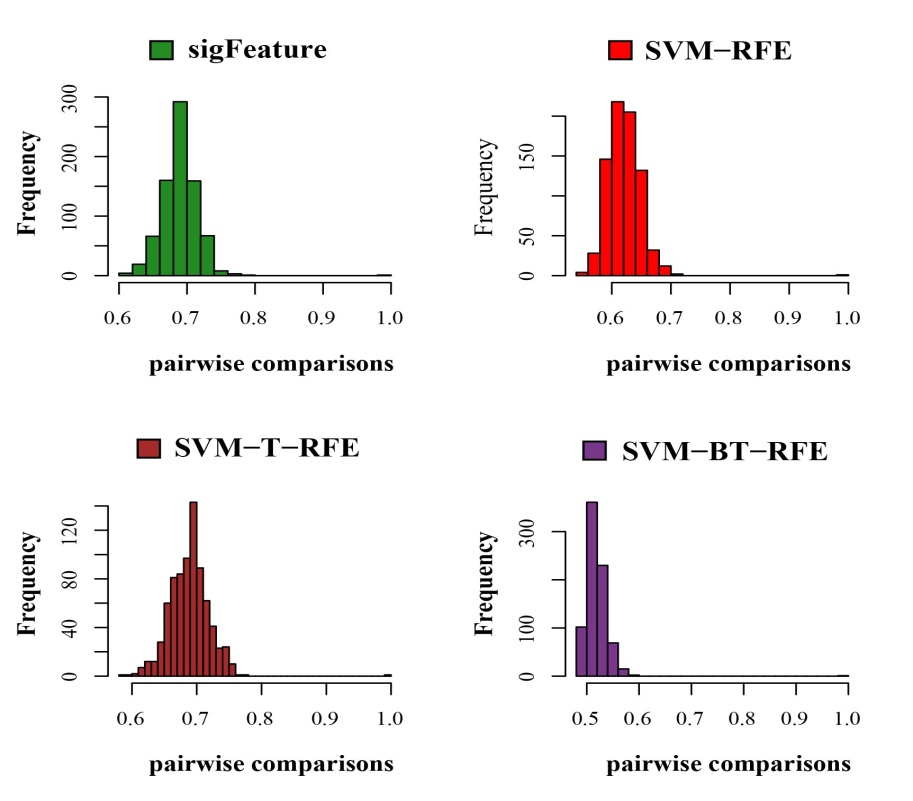


**Figure 3:** **Histogram plots for pair wise stability comparison of the features**. Distribution plots of the pair wise stabilities for the data set GSE119810 where different algorithms produce the feature lists. In each iteration of the algorithms, we used 40 bootstraps, eliminated E = 1% features, used a signature size of 10% and selected the CLA aggregation model.

**2.3. Classification performance result**


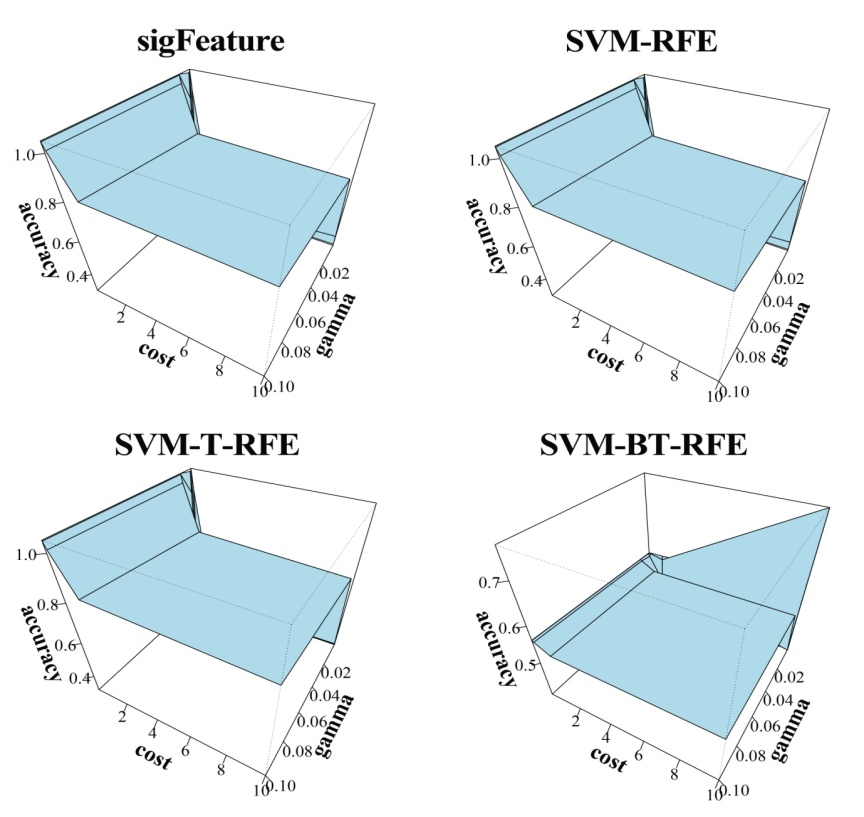


**Figure 4: 3D representation of cost, gamma and classification accuracy.** The Cost and gamma values are selected to determine the best performance in the classification of features selected (top one thousand features by CLA) by different selection algorithms.


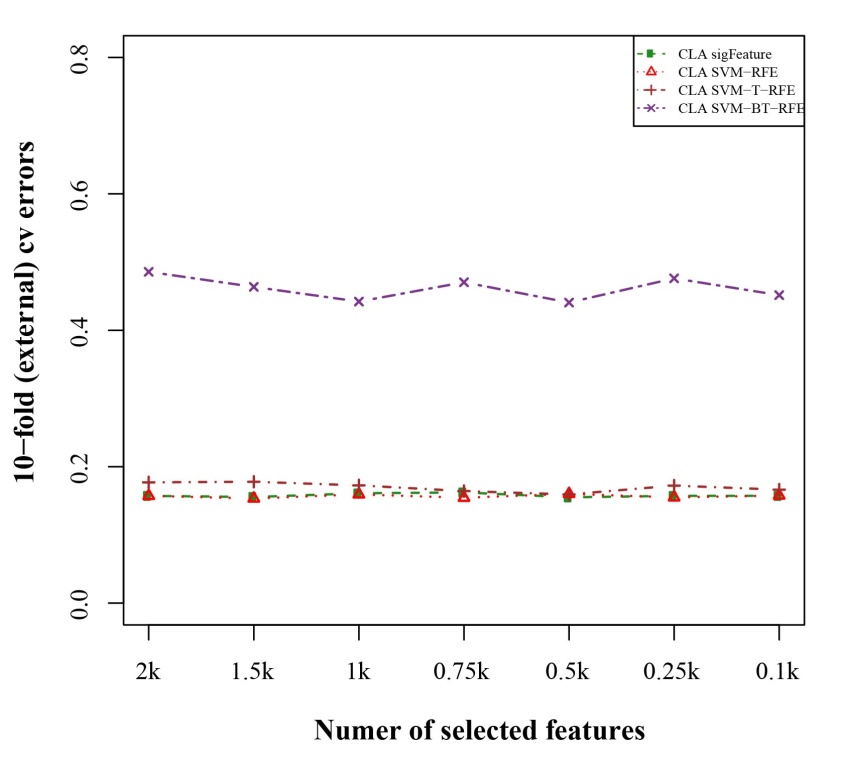


**Figure 5: 10-fold external cross-validation error plot.** The classification performances of the top features are shown here which are selected by different feature selection algorithms. We used 40 bootstraps and eliminated E=1% features.

**2.4. Differentially significant features**


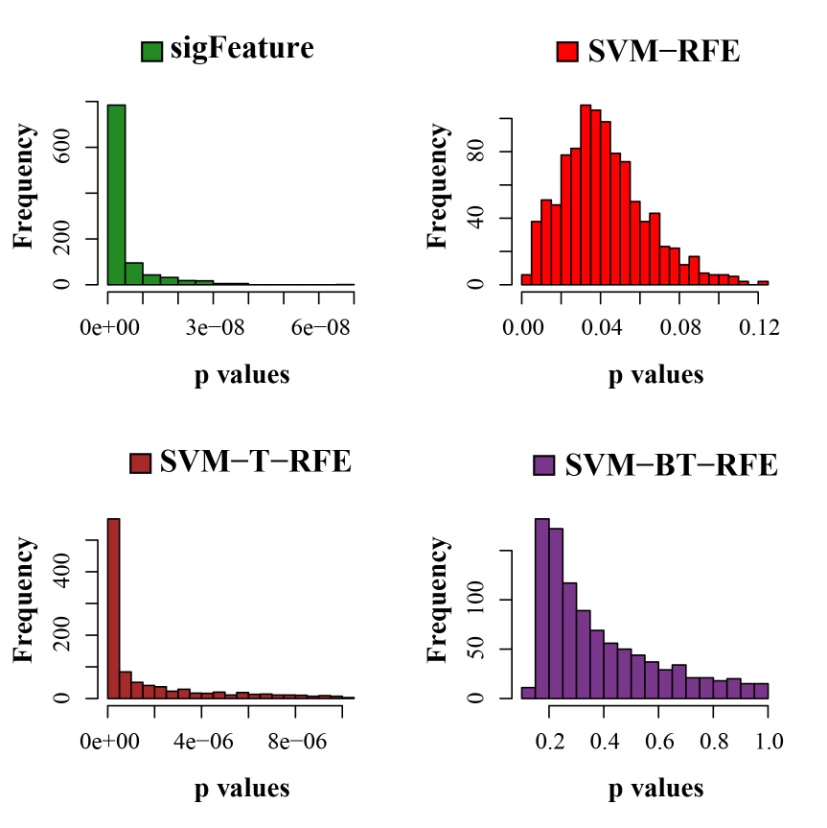


**Figure 6: Histogram plots of unadjusted p-values.** The comparison of the average unadjusted p-values are shown, which are calculated individually using the top one thousand features between classes. The list of features is made using 40 bootstrap sub-sets where the feature selection algorithms remove E = 1% features at each iteration.


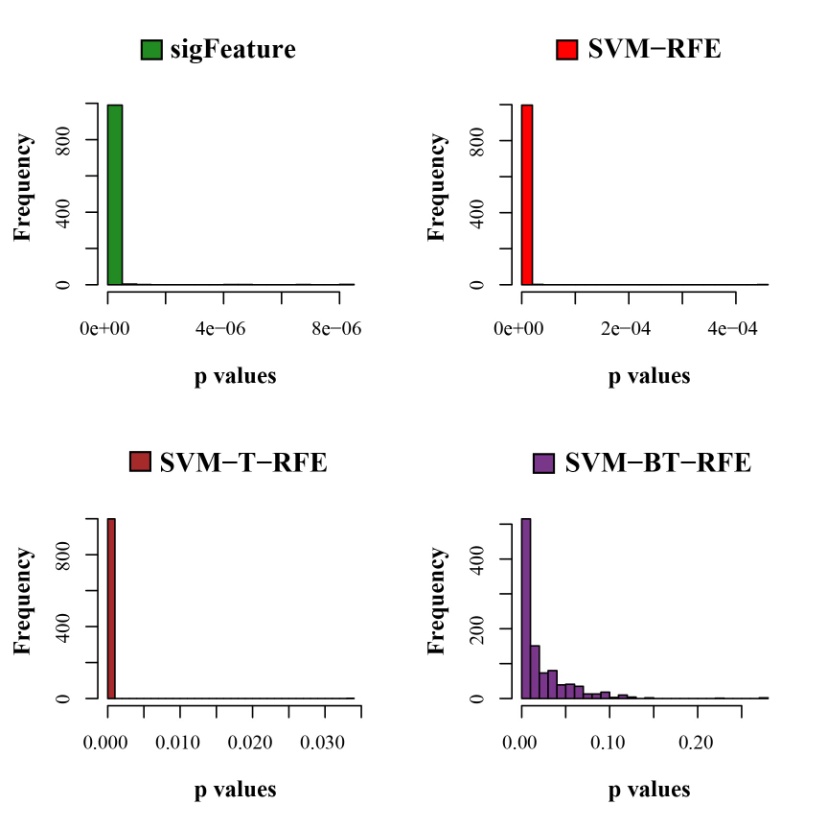


**Figure 7:** **Histogram plots of unadjusted p-values (using top 1000 robust features).** The comparison of unadjusted p-values are shown, which are calculated by using top one thousand features (from the robust feature list) individually between the classes.
